# *Mycobacterium tuberculosis* RipA Dampens TLR4-Mediated Host Protective Response Using a Multi-Pronged Approach Involving Autophagy, Apoptosis, Metabolic Repurposing, and Immune Modulation

**DOI:** 10.3389/fimmu.2021.636644

**Published:** 2021-03-04

**Authors:** Mohd Shariq, Neha Quadir, Neha Sharma, Jasdeep Singh, Javaid A. Sheikh, Mohd Khubaib, Seyed E. Hasnain, Nasreen Z. Ehtesham

**Affiliations:** ^1^Indian Council of Medical Research-National Institute of Pathology, New Delhi, India; ^2^Jamia Hamdard Institute of Molecular Medicine, Jamia Hamdard, New Delhi, India; ^3^Department of Biotechnology, School of Chemical and Life Sciences, Jamia Hamdard, New Delhi, India; ^4^Dr. Reddy's Institute of Life Sciences, University of Hyderabad Campus, Hyderabad, India; ^5^Department of Biochemical Engineering and Biotechnology, Indian Institute of Technology, Delhi (IIT-D) Hauz Khas, New Delhi, India

**Keywords:** autophagy, RipA, peptidoglycan hyrolase, NFkB, TLR4

## Abstract

Reductive evolution has endowed *Mycobacterium tuberculosis* (*M. tb*) with moonlighting in protein functions. We demonstrate that RipA (Rv1477), a peptidoglycan hydrolase, activates the NFκB signaling pathway and elicits the production of pro-inflammatory cytokines, TNF-α, IL-6, and IL-12, through the activation of an innate immune-receptor, toll-like receptor (TLR)4. RipA also induces an enhanced expression of macrophage activation markers MHC-II, CD80, and CD86, suggestive of M1 polarization. RipA harbors LC3 (Microtubule-associated protein 1A/1B-light chain 3) motifs known to be involved in autophagy regulation and indeed alters the levels of autophagy markers LC3BII and P62/SQSTM1 (Sequestosome-1), along with an increase in the ratio of P62/Beclin1, a hallmark of autophagy inhibition. The use of pharmacological agents, rapamycin and bafilomycin A1, reveals that RipA activates PI3K-AKT-mTORC1 signaling cascade that ultimately culminates in the inhibition of autophagy initiating kinase ULK1 (Unc-51 like autophagy activating kinase). This inhibition of autophagy translates into efficient intracellular survival, within macrophages, of recombinant *Mycobacterium smegmatis* expressing *M. tb* RipA. RipA, which also localizes into mitochondria, inhibits the production of oxidative phosphorylation enzymes to promote a Warburg-like phenotype in macrophages that favors bacterial replication. Furthermore, RipA also inhibited caspase-dependent programed cell death in macrophages, thus hindering an efficient innate antibacterial response. Collectively, our results highlight the role of an endopeptidase to create a permissive replication niche in host cells by inducing the repression of autophagy and apoptosis, along with metabolic reprogramming, and pointing to the role of RipA in disease pathogenesis.

## Introduction

*Mycobacterium tuberculosis* (*M. tb*) is an intracellular human pathogen that interacts with macrophages through multiple phagocytic receptors, including pattern recognition receptors. The interaction of its cell wall and protein components to these receptors initiate pro-inflammatory cascade (1–3). Toll-like receptors (TLRs) include TLR2 and TLR4 signals through the NFκB signaling pathway. Activation of these receptors through the engagement of *M. tb* antigens leads to the secretion of pro-inflammatory cytokines, such as TNF-α, IL-6, and IL-12 (4, 5). This activates the innate, and later, adaptive cellular milieu to aid in pathogen clearance along with other antibacterial strategies (6, 7).

Intracellular pathogens have evolved multiple strategies for evading host defense mechanisms. The tactics include inhibition of various pathways like phagosome maturation, antigen processing/presentation, IFN-γ signaling pathway, and autophagy (8, 9). Of late, autophagy has gained a special interest in host-pathogen interaction. Autophagy is a cellular process that maintains intracellular quality control in the face of various stressors that, in normal conditions, play a housekeeping role. Autophagy is a part of both innate and adaptive immunity. Autophagy initiates the formation of new vesicles, i.e., phagophore, which is enlarged, elongated, and generated into a double membrane-bound organelle, the autophagosome. The autophagosome, thereafter, fuses with the lysosome and matures into a phagolysosome for recycling or degradation. It serves as an essential host defense mechanism that can also eliminate invading intracellular bacteria like *Streptococcus, Shigella, Legionella*, and *Salmonella typhimurium*, which was thus termed as xenophagy (10–14). Studies involving autophagy-deficient mice have demonstrated that autophagy protects against active tuberculosis by reducing the bacterial load and inflammation (15, 16). However, to persist inside the host cells, many of the bacterial pathogens have evolved strategies to escape this process of selective host autophagy (17).

Multiple pieces of evidence show that *M. tb* too avoids autophagic degradation and may exploit this process for its advantage by utilizing various effector proteins (18, 19). *M. tb* encodes a wide range of effector molecules that can trigger immune responses or manipulate signaling pathways within the host to promote its persistence (20). *M. tb* antigens, NuoG, ESAT-6, Hsp16.3, and Eis, have been already shown to downregulate the host autophagic process (21–25). Conversely, one of the mycobacterial TLR2 ligand, the 19 kDa lipoprotein, strongly activates autophagy in monocytes and macrophages, whereas Ag85B has been shown to induce autophagy in antigen-presenting cells. It has already been demonstrated that pathogenic *M. tb* can be targeted by selective autophagy through the activation of the ESX-I type VII secretion system (26). Recently, a mycobacterial surface protein PE-PGRS29 has been implicated in recruiting autophagy receptor p62 to deliver mycobacteria into LC3-associated autophagosomes (27). These reports indicate a diverse role of the autophagy process in mycobacterial virulence (28).

RipA (Rv1477) is an essential endopeptidase that acts as a peptidoglycan hydrolase. Previously, we have shown that RipA requires the ATP-dependent chaperone, MoxR1, for its secretion through the twin-arginine secretion system (29). It has been proposed that after secretion, RipA interacts with mammalian cell entry protein, Mce2B, and may, therefore, be targeted to the host macrophage. As RipA is a secretory protein, *M. tb* may utilize secreted RipA to modulate host–pathogen interaction, as a moonlighting function (30), besides its enzymatic role (31). However, the role of RipA in immune-modulation and host–pathogen interaction remains unexplored. The *in-silico* analysis revealed an LC3 interacting motif in RipA, which suggested its role in autophagic modulation. In the present study, we report that RipA inhibits cellular autophagy in RAW264.7 cells by the activation of PI3K-AKT-mTORC1 signaling axis mediated by the innate immune receptor TLR4. We show that RipA induces the expression of macrophage activation markers and the secretion of pro-inflammatory cytokines through the activation of TLR4. We also show that RipA localizes to the mitochondria and regulates the levels of mitochondrial enzymes to induce metabolic reprogramming of macrophages. It also inhibits caspase-dependent programmed cell death to favor pathogen survival. Our findings provide mechanistic insights into the strategies adopted by *M. tb* to manipulate host functions to ultimately assist bacterial infection.

## Materials and Methods

### Cell Culture and Growth Conditions

The mouse macrophage ΔTLR1, ΔTLR2, ΔTLR4, ΔTLR6, ΔMyd88, and ΔMyd88/TRIF knockout cell lines were obtained through BEI Resources Repository (NIAID, NIH, USA). These cell lines, as well as RAW264.7 and human epithelial cell line HEK293T, were grown in the Dulbecco's Modified Eagle's medium (DMEM) medium (Gibco, Invitrogen). The cell culture medium was supplemented with 10% fetal bovine serum (Gibco) and 1% antibiotic-antimycotic solution (Gibco), and cells were grown and maintained under the standard tissue culture conditions of 37°C and 5% CO_2_. All experiments in the various cell lines were performed within 8 passages after the seeding of the original frozen stocks.

### Molecular Cloning, Expression, and Purification of RipA (*ripA*) Gene

The cloning of *ripA* in pET28a and pST-2K plasmid has been described previously (29). Briefly, the *ripA* gene was cloned in *Escherichia coli* and mycobacteria shuttle vector pST-2K to study its function in native condition (32). The expression of RipA in recombinant *Mycobacterium smegmatis* was verified by Western blot analysis using RipA specific anti-serum (Supplementary Figure 2E). For cloning in EGFP-N1 and pcDNA3.1^+^ plasmids, the *ripA* gene was amplified using *M. tb* H_37_Rv DNA as a template and inserted between *Eco*RI-*Kpn*I, and *Hin*dIII-*Not*1 restriction enzyme sites. Primer pairs, plasmids, and bacterial strains used are given in Supplementary Tables 2, 3. The plasmids were transformed into *E. coli* BL21 (DE3) cells for recombinant protein expression and purification (pET28a-RipA), and into DH5α for plasmid maintenance and propagation for transfection studies (EGFP-N1-RipA and pcDNA3.1^+^-RipA). Briefly, for RipA overproduction, the recombinant protein expression was induced with 1 mM IPTG (MP Biomedicals, USA) for 3 h at 37°C in Luria-Bertani (LB) broth containing kanamycin 50 μg/ml. Protein purification was carried out as described earlier (29). The purity of recombinant RipA was checked by using SDS-PAGE and Western blot analysis, which revealed 2 bands that correspond to unprocessed (along with signal sequence) and processed form (signal sequence cleaved) (Supplementary Figures 2A,B). Possible endotoxin contamination was removed by incubating the purified protein with polymyxin-B agarose beads (Sigma, USA) as described previously (33). The endotoxin content of the purified recombinant RipA was measured using the limulus amebocyte lysate assay kit (Pierce™ LAL Chromogenic Endotoxin Quantitation Kit, Thermo Fisher, USA). No detectable amount of endotoxin contamination was observed in the protein fractions incubated with Polymyxin-B agarose beads.

### Cytokines Estimation From Culture Supernatants of RAW264.7 and TLR Mutant Mouse Macrophage Cells

RAW264.7, ΔTLR1, ΔTLR4, ΔTLR2, ΔTLR6, ΔMyd88, and ΔMyd88/TRIF mouse macrophage cells were seeded (~1 × 10^6^ cells per well) in a 12-well-culture plate and left for 2 h at 37°C for adherence. After adherence, cells were treated with various concentrations of recombinant RipA protein (0.5, 1, and 2 μg/ml) or lipopolysaccharide (LPS) (1 μg/ml; positive control) (Sigma, USA). The protein treatment dose was pre-standardized for optimal release of cytokines and other cellular markers. Heat-inactivated (HI) RipA protein-treated cells were used as negative controls for cytokine production. After 24 and 48 h of treatment, supernatants were collected and stored at −80°C until used. The estimation of pro-inflammatory cytokines, such as TNF-α, IL-6, and IL-12, and anti-inflammatory cytokine, IL-10, was performed using mouse ELISA kit (BD Biosciences, San Jose CA, USA) following the manufacturer's instructions.

### Flow Cytometry Analysis

The surface expression of macrophage activation markers, such as MHC-II, CD80, CD86, and TLR4, was determined using RAW 264.7 cells (0.5 × 10^6^) treated with various concentrations of RipA (1 and 2 μg/ml). Cells were seeded in 24-well-culture plates. After 2 h of seeding, cells were treated with RipA, harvested after 24 h, and incubated with anti-mouse Alexa Fluor 488-MHCII, PE-CD80, APC-CD86, and PE-TLR4. The samples were processed as per protocol provided by the supplier (BD Biosciences, San Jose CA, USA). LPS-treated cells (100 ng/ml) were used as a positive control for the TLR4 expression. For Annexin V/PI apoptosis assay, 0.5 million cells were seeded in a 24-well-culture plate. After 2 h of adherence, cells were treated with various concentrations of RipA (1 and 2 μg/ml) and then harvested after 24 h. Staurosporine-treated cells (0.1 μM, Sigma, USA) were used as a positive control. About 2 μM pan-caspase inhibitor Z-VAD-FMK (MP Biomedicals, USA) was used for 24 h. The samples were processed as instructed by the manufacturer of the assay kit (BD Biosciences, San Jose CA, USA). Fluorescence intensity was measured using the BD FACSVerse flow cytometer (BD Biosciences, San Jose, CA, USA). Untreated and HI RipA-treated samples were taken as negative controls in both the assays. The data were analyzed by FlowJo software (Tree Star Inc., USA).

### Western Blot Analysis

For Western blot analysis, ~2 million RAW264.7 cells and ΔTLR4 knockout were seeded in 6-well-culture plates and treated with RipA (1 and 2 μg/ml) after 2 h of seeding. A separate set of the same number of cells was infected with *M. smegmatis* (wild type, RipA transformed, and vector transformed; MOI-1:10) after 12 h of seeding. After 24 h of treatment, cells were harvested in 2.5X-SDS loading buffer (25% glycerol, 0.125 M Tris-HCl, pH 6.8, 5% SDS, 0.1% bromophenol blue, with 100 mM dithiothreitol added fresh each time). An equal volume of protein samples was loaded in SDS-PAGE and transferred onto a polyvinylidene difluoride (PVDF) membrane. These were incubated with primary antibodies against RipA (1:4,000), NFκB1, Caspase 3 (Cloud-Clone Corp, USA), P-65 (ThermoFisher Scientific, USA), LC3B, P62/SQSTM1, Beclin 1, Rab7, pPI3K, pAKT, pmTORC1, pMDM2 (Mouse double minute 2 homolog), pULK1, pJNK 1/2, tJNK 1/2 cytochrome C, CoxIV, succinate dehydrogenase, pyruvate dehydrogenase, HSP60, PDI (Protein disulfide isomerase), BIP (Immunoglobulin heavy-chain-binding protein) (Cell Signaling, USA), glyceraldehyde 3-phosphate dehydrogenase (GAPDH), and β-actin antibodies (Santa Cruz Biotechnology Inc., USA). HRP conjugated secondary antibodies (anti-mouse or anti-rabbit) were used for signal generation (Sigma, USA). For studying cellular autophagy in the presence of rapamycin and bafilomycin A1 (Sigma, USA), macrophage cells were treated with 200 nM rapamycin and 50 nM bafilomycin A1 for 6 h in the presence of RipA. PVDF membranes were developed by enhanced chemiluminescence substrate ECL (DSS Takara Bio India Pvt. Ltd.). Images were captured using the BioRad ChemiDoc MP imaging system (Bio-Rad Laboratories India Pvt. Ltd.). The band intensity was quantified densitometrically by using ImageJ software (34) and normalized to GAPDH or β-actin (Santa Cruz Biotechnology Inc., USA).

### Immunofluorescence Microscopy

HEK293T cells were grown in 24-well-tissue culture plates on sterile poly-L lysine coated glass coverslips (Sigma, USA). Cells were transfected with pcDNA3.1^+^, pcDNA3.1^+^-RipA, EGFP-N1, or EGFP-N1-RipA using Lipofectamine 3,000 Transfection Reagent (ThermoFisher Scientific, USA). After 24, 48, and 72 h of transfection, cells were treated with 300 nm of MitoTracker Deep Red FM (ThermoFischer Scientific, USA) for 45 min in incomplete DMEM. In another experiment, RAW264.7 cells were treated with RipA (0.5, 1, and 2 μg/ml) for 24 h. After fixation and permeabilization, cells were blocked and coverslips were incubated with anti-RipA (1:500), and various other primary antibodies (anti-LC3B, anti-NFkB, anti-CoxIV, and anti-TLR4) as desired. Alexa Fluor 488 and Alexa Fluor 594-conjugated secondary antibodies were added at 1:500 dilution for 2 h at room temperature. Finally, coverslips were sealed with ProLong Glass Antifade mountant (Thermo Fisher, USA). Samples were visualized at 60 and 63X through Nikon's Confocal microscope and Carl Zeiss Fluorescence microscope equipped with oil immersion objectives. Images were captured and processed using NIS Elements 5.21.00 and an Axio Cam Hrm digital camera and Axio-Vision-4.8 software. Punctate foci representing LC3 were counted manually from 10 different fields and data presented are a representative count from 50 cells.

### Structural Modeling of RipA and RipA-TLR4 Complex

The amino acid sequence of *M. tb* RipA (O53168) was retrieved from the UniProtKB database. The retrieved amino acid sequence was subjected to a protein–protein BLAST (BLASTp) search against the Protein Data Bank (PDB) to identify suitable template structures for comparative modeling. The 3D structure prediction was performed using i-Tasser (35–37). The resultant *z*-scores were analyzed to check the likelihood that the domains had correct folds and tertiary structure. The final model was also checked for its quality using Ramachandran plot and ProSA analysis. Further, the modeled structure was subjected to molecular dynamic (MD) simulations for 100 ns before docking. For the modeling of (TLR4)_2_-(RipA)_2_ heterotetramer, the previously modeled RipA as a ligand was docked onto the TLR4 dimer (TLR4)_2_, (PDB id: 3vq2) receptor using the ClusPro 2.0 protein–protein docking engine (38–40).

### MD Simulations

Molecular dynamic simulation of (TLR4)_2_ alone and (TLR4)_2_-(RipA)_2_ were performed using GROMACS 5.1 and GROMOS 54a7 all-atom force field, employing periodic boundary conditions (41–43). The starting models were solvated in a periodic box with an SPC/E water model and with 10 nm spacing from each edge of protein. Counter ions were added to neutralize and maintain the ionic concentration of 0.1 M in both systems. Subsequently, these were energy minimized using the steepest descent protocol followed by an equilibration run of 1 ns for both NVT and NPT ensembles with restraints on the protein atoms. Systems were simulated at 310K maintained by a Berendsen thermostat and pressure coupling employing a Parrinello-Rahman barostat using a 1 bar reference pressure with the compressibility of the 4.5e^−5^ bar using the isotropic scaling scheme. Electrostatic interactions were calculated using the Particle Mesh Ewald (PME) summation with a two-fs time step for each run of 50 ns. The resultant trajectories were analyzed using standard GROMACS tools. Images were constructed using PyMol.

### Principal Component Analysis (PCA)

For removing high-frequency background motions from simulation trajectories, PCA was performed to collect low amplitude dynamics. Briefly, it was done by calculating and diagonalizing the mass-weighted covariance matrix for the C-α atoms of the (TLR4)_2_ alone and in the compound state with RipA. The resultant trajectories were projected onto the first eigenvector, and the fluctuations were calculated between extreme projections. Finally, porcupine plots were generated using PyMol.

### Computational Analysis of RipA Sequence Information

The RipA protein sequence was analyzed for its antigenic potential and the presence of B cell epitopes using the Scratch Protein Predictor (http://scratch.proteomics.ics.uci.edu/) and B Cell Epitope Prediction Tools (http://tools.iedb.org/main/bcell/). Mitochondrial targeting sequence in RipA was predicted using MITOPROT (https://ihg.gsf.de/ihg/mitoprot.html), TargetP 1.1 (http://www.cbs.dtu.dk/services/TargetP/), and PSORT II (https://psort.hgc.jp/form2.html). The presence of functional LC3 interacting region motifs in RipA was predicted using iLIR (http://repeat.biol.ucy.ac.cy/cgi-bin/iLIR/iLIR_cgi).

### Statistical Analysis

The Student's *t*-test was used for the analysis of the results wherever required. The data were represented as the mean of replicates ± SD. *p* < 0.05 was considered as statistically significant. Data analysis was carried out using Microsoft Excel software.

## Results

### RipA Induces Macrophage Activation by the Enhanced Expression of Surface Markers and Stimulates the Production of Pro-inflammatory Cytokines

**Figure 1:**
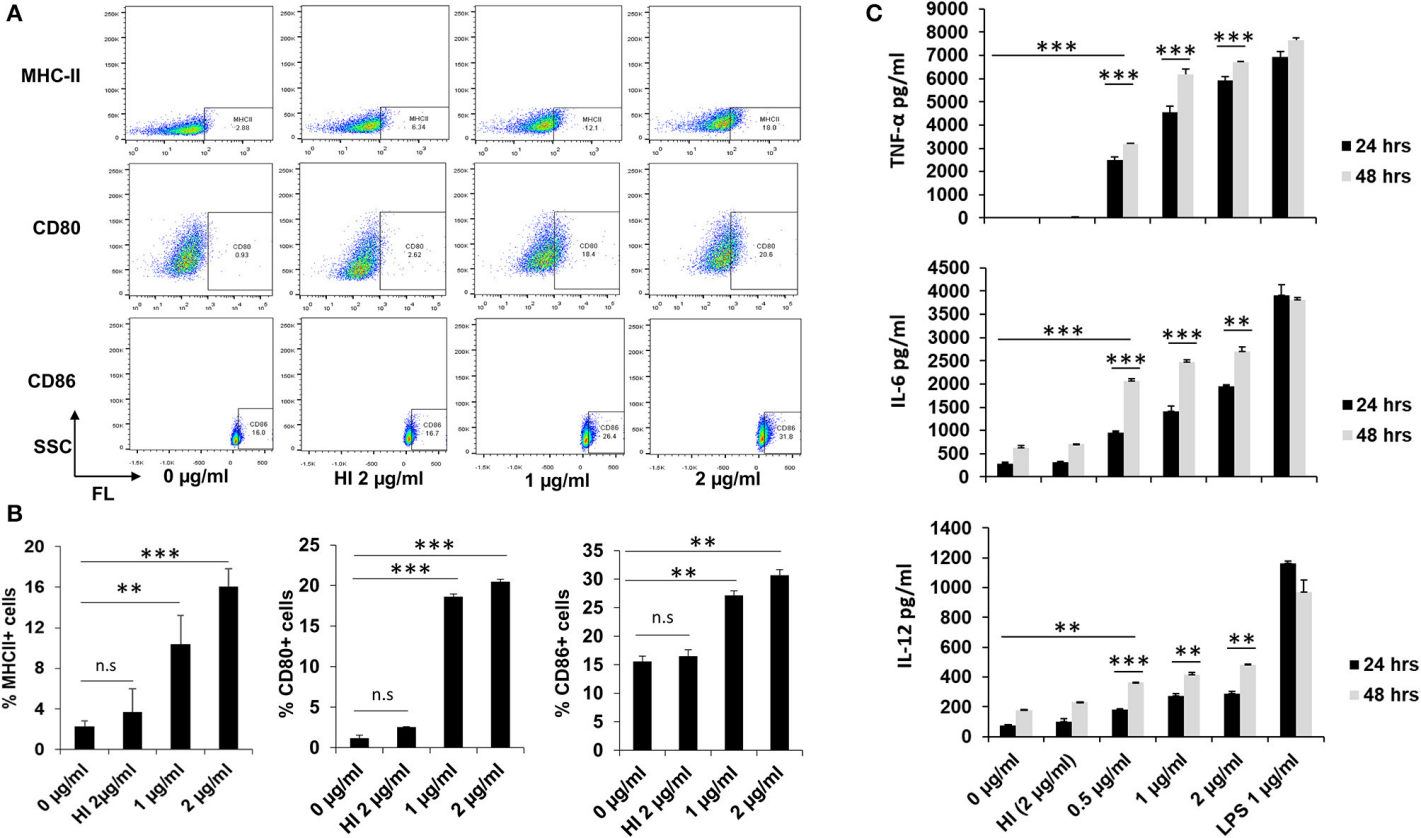
RipA enhances the expression of macrophage activation markers and induces pro-inflammatory cytokines secretion. **(A)** The enhanced expression of surface markers of RAW264.7 cells (MHC-II, CD80, and CD86) at 24 h after treatment with medium alone, 1 μg/ml RipA, 2 μg/ml RipA, and HI RipA. The expression level was determined by FACS analysis using A488, PE, and APC-linked monoclonal antibodies, respectively. Purified RipA was treated with polymyxin B agarose for the removal of endotoxin, and 1 aliquot was digested with proteinase K, followed by heat inactivation at 100°C for 4 h. **(B)** Quantitative representation of the expression of MHC-II, CD80, and CD86 on the surface of RAW264.7 cells. **(C)** RAW264.7 cells were treated with RipA (0.5, 1, and 2 μg/ml), LPS 1 μg/ml, and HI RipA (2 μg/ml). After 24 and 48 h of treatment, cell culture supernatants were collected, followed by quantification of TNF-α, IL-6, and IL-12 levels by sandwich ELISA. LPS was used as a positive control for the induction of pro-inflammatory cytokines. Untreated and HI treated cells were used as negative controls. Data are representative of 3 independent experiments (3 replicates per experiment) and expressed as means ± SD. **p < 0.01, and ***p < 0.001 vs. controls.

Our *in silico* computational analysis revealed that RipA has comparable antigenic properties to known secretory antigens of *M. tb*, such as ESAT-6, CFP-10, and PST-S1 (Supplementary Figures 1A,B). Therefore, we were interested in deciphering whether recombinant RipA (Supplementary Figures 2A–E) can modulate the functional capability of macrophages. In the absence of any information regarding *in-vitro*/*in-vivo* physiological concentrations of RipA, we pre-standardized an optimal range of concentrations for the treatment of RAW264.7 cells (0.5, 1, and 2 μg/ml). This range of concentration was non-toxic for cells that led to optimum (minimal to saturation) release of cytokines and expression of other cellular markers. Treatment of macrophage cells with endotoxin-free RipA protein-induced enhanced surface expression of antigen-presenting MHC-II molecules along with co-stimulatory molecules, CD80 and CD86, in a concentration-dependent manner (Figures 1A,B). The observed changes in the expression were most significant for CD80 and MHC-II (*P* < 0.001), followed by the CD86 molecule (*p* < 0.01). A similar enhanced expression of MHC-II and CD80 was observed when phorbol 12-myristate-13-acetate differentiated THP-1 cells were treated with RipA (Supplementary Figure 2F). Moreover, to analyze the effect of RipA on macrophages, we quantitated pro-inflammatory and anti-inflammatory cytokines by treating macrophage cells with various concentrations of RipA (0.5, 1, and 2 μg/ml). RipA-stimulated RAW264.7 cells secreted high levels of TNF-α and IL-6 and moderate levels of IL-12 in a dose- and time-dependent manner. In contrast, untreated or HI RipA treated cells produced negligible amounts of these cytokines (Figure 1C). We did not observe any significant change in the secretion profile of IL-10. These results demonstrate that RipA elicits the secretion of pro-inflammatory cytokines, TNF-α, IL-6, and IL-12, by macrophages in a dose- and time-dependent manner.

### RipA Activates Canonical NFκB Signaling Pathway

**Figure 2:**
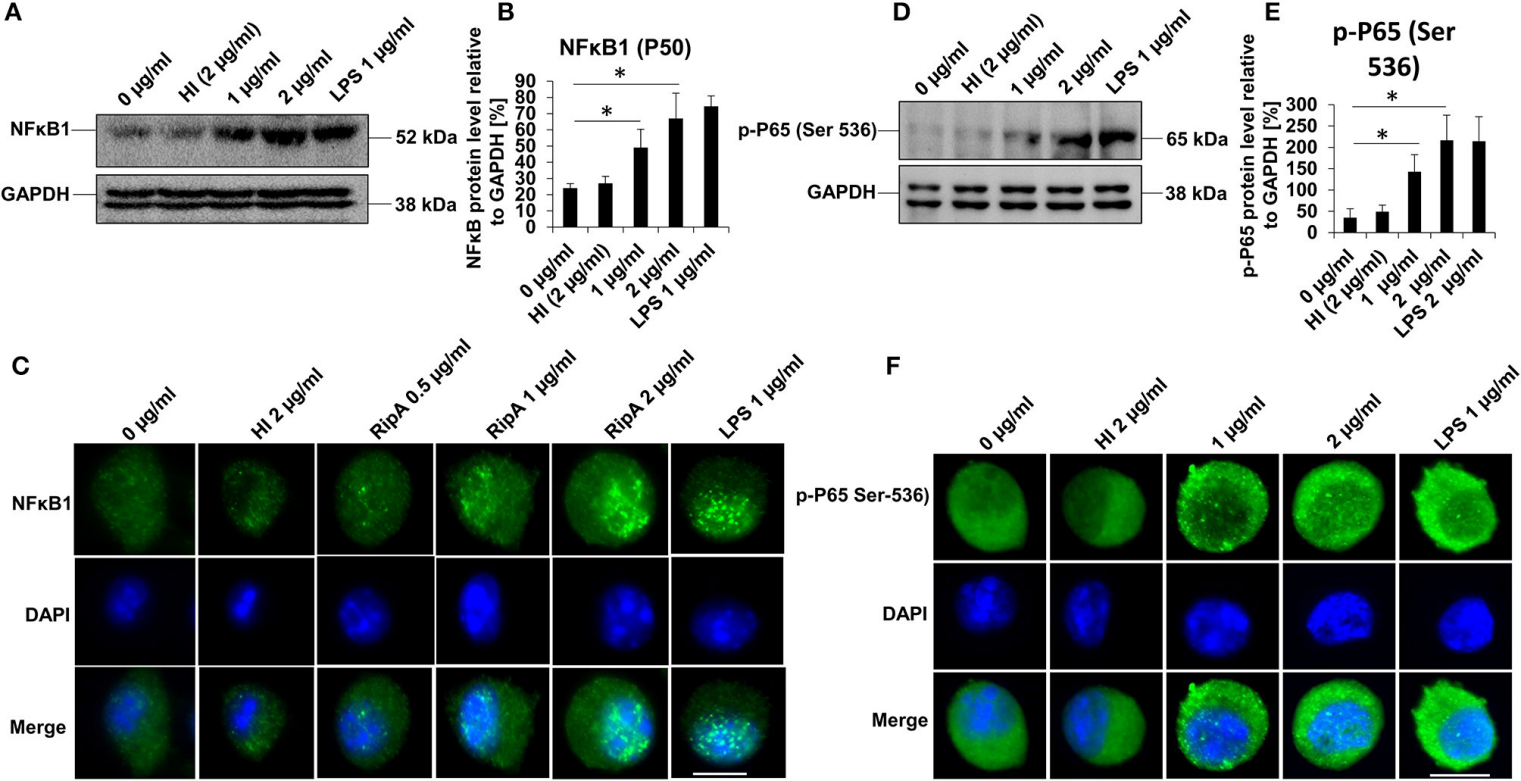
RipA activates the canonical NFκB signaling pathway. **(A)** Western blots showing the expression level of NFκB1 (P-50) subunit in RipA-treated RAW264.7 cells. RAW264.7 cells were treated with RipA (1 and 2 μg/ml) for 24 h. The size and positions of the bands are marked on the figure. **(B)** Densitometric analysis of the P-50 subunit of NFκB. P-50 protein level was expressed relative to glyceraldehyde 3-phosphate dehydrogenase (GAPDH) [%]. **(C)** Immunofluorescence microscopic images depicting nuclear translocation of P-50. Concentrations of RipA and antibody used are marked on the figure. **(D)** Western blots showing the level of the phosphorylated pP-65 subunit of NFκB in RipA treated RAW264.7 cells. **(E)** Densitometric analysis of pP-65 subunit. The pP-65 level was expressed relative to GAPDH [%]. **(F)** Immunofluorescence microscopic images showing nuclear translocation of phosphorylated P-65. DAPI was used to stain the nucleus. Untreated and HI treated cells were used as negative controls. Lipopolysaccharides (LPS) treatment was used as a positive control. GAPDH was used as a loading control. Data are representative of 3 independent experiments and quantification plot expressed as means ± SD. *p < 0.05 vs control. A488 conjugated secondary antibody was used for signal generation. Scale bar indicates 10 μm.

Activation of the canonical NFκB pathway is essential in the production of pro-inflammatory cytokines (44). We accordingly analyzed the activation status of NFκB in response to RipA treatment. RAW264.7 cells were treated with purified recombinant RipA protein at various concentrations (0.5, 1, and 2 μg/ml). The expression and nuclear translocation of NFκB1 (P-50) along with phosphorylation (Ser536) and nuclear translocation of the RelA (P-65) subunit were analyzed. As shown in Figures 2A,B, the level of the P-50 subunit increased with an increase in the concentration of RipA, indicating an enhanced activation of pro-inflammatory response. Immunofluorescence microscopy revealed that RipA-induced nuclear translocation of the P-50 subunit in a dose-dependent manner which suggested its activation (Figure 2C). P-50 and P-65 subunits form the most common heterodimeric activated form of NFκB (45). Also, IKK-β mediated phosphorylation at Ser536 of the P-65 subunit is critically essential for the activation of the canonical NFκB pathway (45). We, therefore, looked at the phosphorylation of the P-65 subunit by Western blot analysis and its nuclear translocation by immunofluorescence microscopy. RipA elicited the phosphorylation of the P-65 subunit as evident from the concentration-dependent increase in its phosphorylation status (Figures 2D,E). It also induced increased nuclear translocation in a dose-dependent manner which is suggestive of its activation status (Figure 2F). These findings show that RipA-induced enhanced expression and activation of canonical NFκB subunits to maintain sustained activation of the pro-inflammatory response. These observations, thus, suggest the involvement of RipA-mediated NFκB signaling in macrophage activation and secretion of pro-inflammatory cytokines.

### RipA Induces Overexpression and Membrane Organization of TLR4 in Macrophage Cells

**Figure 3:**
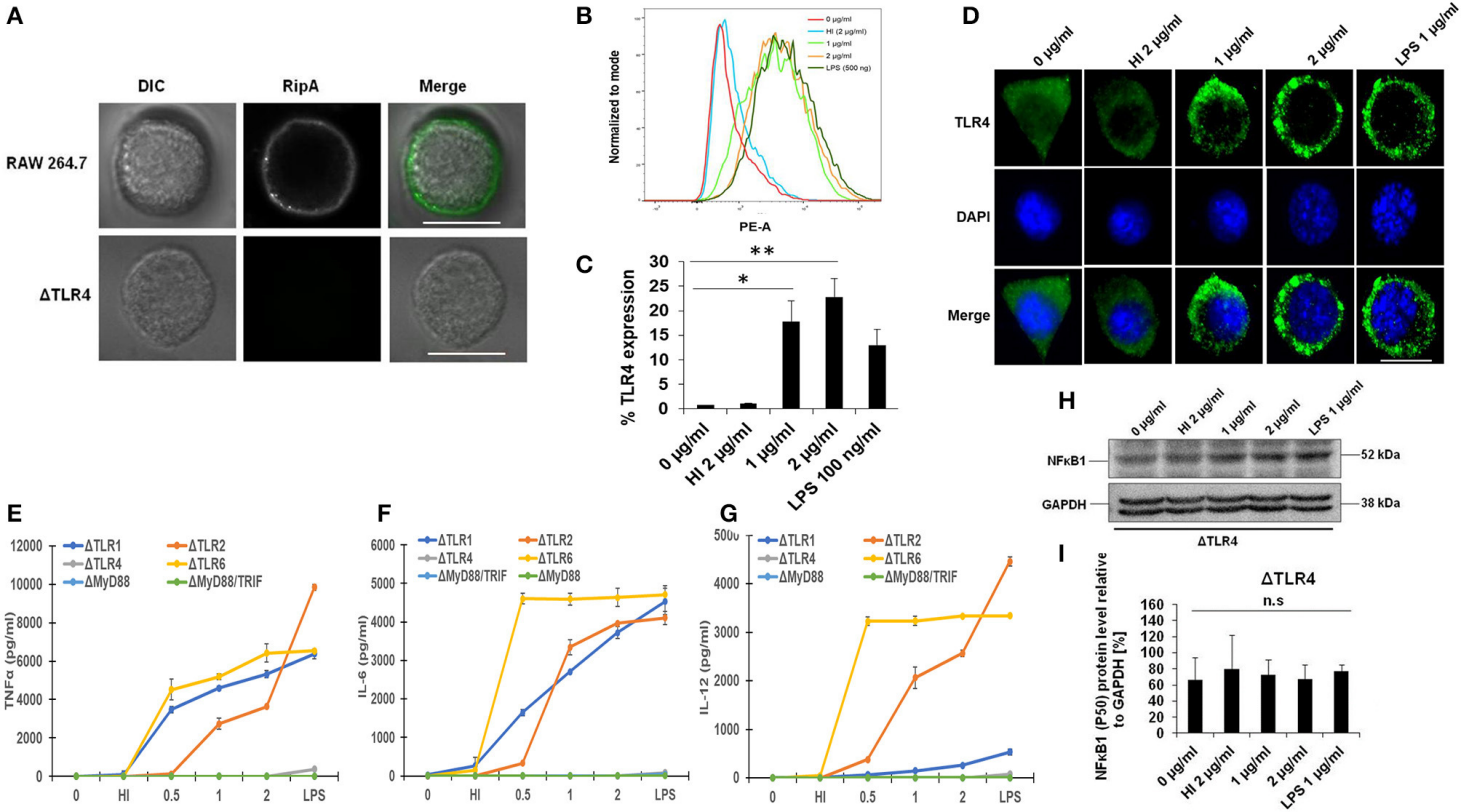
RipA induces enhanced expression of toll-like receptor (TLR)4 in treated macrophage cells. **(A)** RAW264.7 and ΔTLR4 cells were treated with purified RipA (2 μg/ml). After 2 h of treatment, cells were fixed with 4% formaldehyde to freeze the interaction of RipA to the cell surface localized receptor. Cells were washed and probed with polyclonal anti-RipA antibody (1:500) for 2 h. After treatment with primary antibody, cells were probed with anti-rabbit A488 conjugated secondary antibody, mounted on ProLong Anti Fade Glass mountant, and visualized using Carl Zeiss fluorescence microscope. Scale bar indicates 10 μm. **(B)** FACS analysis was used to study the expression level of TLR4 on RipA-treated macrophage cells. Monoclonal anti-TLR4 antibody conjugated to PE fluorophore was used in the FACS experiment. **(C)** The quantification of TLR4 expression is shown as a bar graph. Data are representative of 3 independent experiments (3 replicates per experiment) and quantification plot expressed as means ± SD. *P < 0.05 and **P < 0.01 vs. controls. **(D)** Immunofluorescence microscopic images showing membrane organization and expression of TLR4 in RipA treated macrophage cells. The anti-rabbit TLR4 monoclonal antibody was used to probe the localization of TLR4. A488 conjugated secondary antibody was used for signal generation. DAPI was used to mark the positions of the nucleus. Untreated and heat-inactivated (HI) treated cells were used as negative controls. LPS treated cells were used as a positive control. Scale bar indicates 10 μm. Mouse macrophage knockout cells ΔTLR1, ΔTLR4, ΔTLR2, ΔTLR6, ΔMyd88, and ΔMyd88/TRIF were treated with RipA (0.5, 1, and 2 μg/ml). LPS 1 μg/ml and HI RipA (2 μg/ml) were treated with proteinase K (PK; 50 mg/ml), followed by heat inactivation at 100°C for 4 h served as controls. After 24 h of treatment, cell culture supernatants were collected and TNF-α, IL-6, and IL-12 levels were measured by sandwich ELISA. **(E–G)** TNF-α, IL-6, and IL-12 secretion by macrophages derived from ΔTLR1, ΔTLR4, ΔTLR2, ΔTLR6, ΔMyd88, and ΔMyd88/TRIF mice knockout cell lines after 24 h of treatment as measured by ELISA **(H,I)**. Western blot showing the level of NFkB and its densitometric quantification in ΔTLR4 cells. Data are representative of 3 independent experiments (3 replicates per experiment) and expressed as means ± SD. *p < 0.05 and **p < 0.01 vs. controls.

Macrophages organize the primary innate immune defense against *M. tb* infection using TLRs and play an essential role during the early immune response (46). To determine the specific binding of RipA to TLR4, we first investigated whether RipA exerts its effect through this cell surface localized receptor. For this purpose, we treated RAW264.7 cells with purified recombinant RipA and froze the interaction by chemical cross-linking. Surface localization of RipA was visualized by probing the location of RipA using the anti-RipA antibody. As shown in Figure 3A, RipA was found on the cell surface of treated macrophage cells. On the contrary, there was no signal present when we used TLR4 knockout mouse cell line. We also observed that RipA treatment of macrophage cells induced an enhanced surface expression and membrane organization of TLR4 in a concentration-dependent manner (Figures 3B–D). These findings suggest that RipA interacts with a surface immune receptor TLR4 and induces its enhanced expression to initiate downstream signaling cascades.

### RipA Induces Production of Pro-inflammatory Cytokines by Macrophages in a TLR4 and Myd88 Dependent Manner

Our observation that RipA induces the secretion of pro-inflammatory cytokines by macrophages (Figure 1C) prompted us to explore whether this process is TLR4 dependent. To confirm this, we treated mouse macrophage ΔTLR1, ΔTLR2, ΔTLR4, ΔTLR6, ΔMyd88, and ΔMyd88/TRIF knockout cells with RipA (0.5, 1, and 2 μg/ml). RipA significantly stimulated the secretion of pro-inflammatory cytokines by ΔTLR1, ΔTLR2, and ΔTLR6 mouse macrophage knockout cells, whereas no detectable levels of these cytokines were observed in ΔTLR4, ΔMyd88, and ΔMyd88/TRIF cells (Figures 3E–G). These findings implicate that RipA recognition by TLR4 can trigger macrophages to secrete TNF-α, IL-6, and IL-12. Next, we investigated whether the activation of the NFκB signaling pathway also depends on TLR4 by analyzing the level of the P-50 subunit. We performed the Western blot analysis of the cell lysates prepared from the ΔTLR4 mouse macrophage cells treated with RipA. Western blot analysis showed no significant change in the expression pattern of the P-50 subunit of NFκB (Figures 3H,I). These results suggest that RipA activates the canonical NFκB signaling pathway in RAW264.7 cells *via* TLR4 activation.

### *In-silico* Analyses Reveals That RipA Interacts With the Ligand-Binding Pocket of TLR4

**Figure 4:**
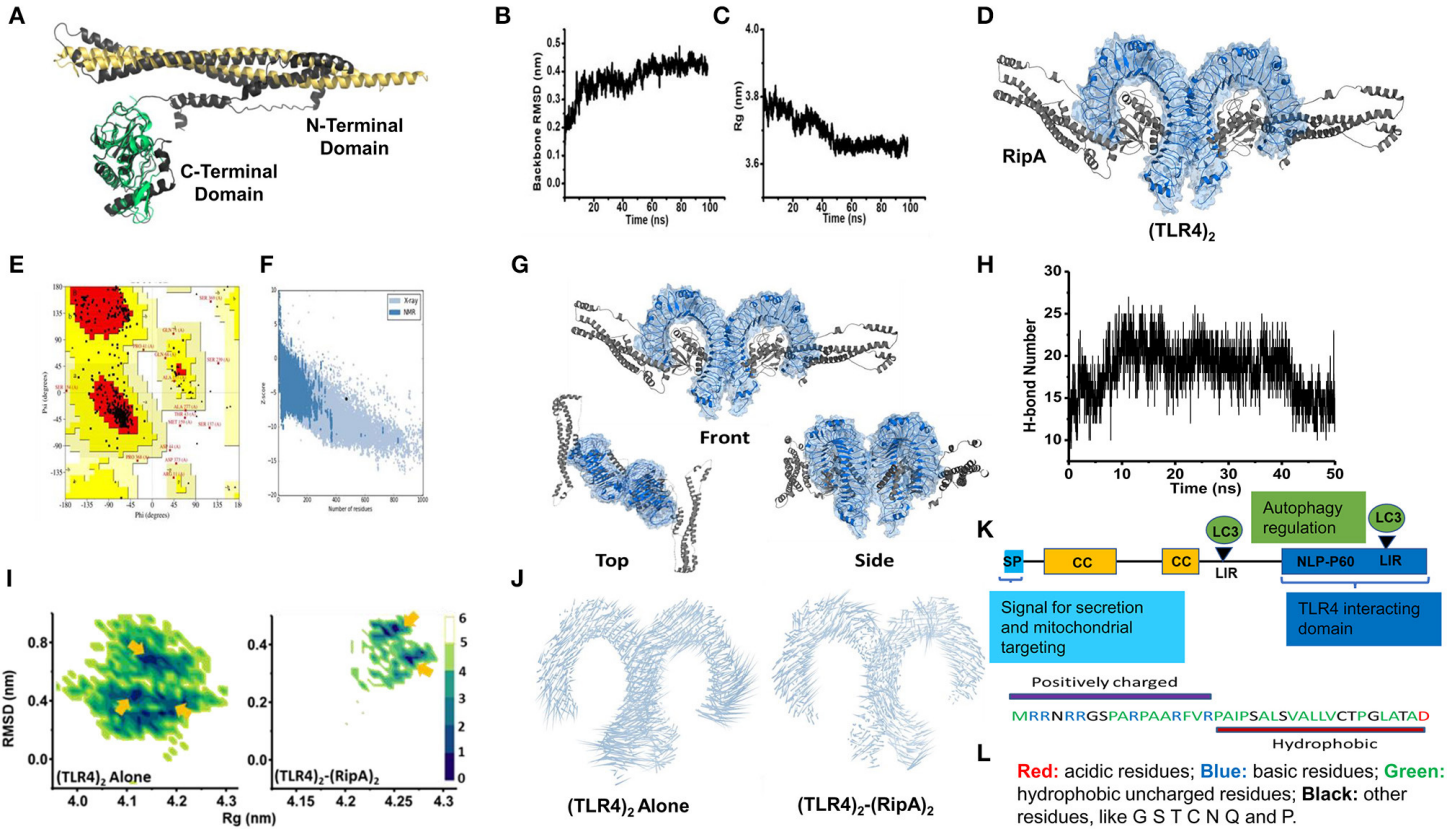
C-terminal domain of RipA interacts with the ligand binding pocket of TLR4 dimer. **(A)** RipA monomeric unit (dark gray) showing its N- and C-terminal domains and aligned structurally with its available individual crystal structures of N-terminal domain (Golden, PDB id: 6ewy) and C-terminal domain (Green, PDB id: 3pbc). **(B,C)** Variations in backbone RMSD **(B)** and gyration radius (Rg) **(C)** during 100 ns simulation of RipA alone. Marginal changes in RMSD and Rg indicate a stable architecture for further experiments. **(D)** (TLR4)_2_-(RipA)_2_ hetero-tetrameric model of the top-scoring structure obtained through ClusPro protein-protein docking. **(E)** Ramachandran plot of the RipA model. **(F)** ProSa based residue-wise energy estimation of the RipA model. **(G)** Top scoring protein-protein docking model obtained from ClusPro docking of TLR4 dimer receptor with RipA. Docking results showed predominant interaction of the C-terminal domain of RipA within the ligand-binding pockets of TLR4 dimer. **(H)** Variation in H-bond network between RipA and ligand-binding pocket of TLR4. **(I)** Free energy landscapes (KJ/mol) projected as a function of 2 principal macroscopic components Rg and backbone RMSD for (TLR4)_2_ alone and (TLR4)_2_-(RipA)_2_. Arrows indicate multiple low free energy basins corresponding to metastable states achieved by (TLR4)_2_ in both sets of simulations. **(J)** Porcupine plots deduced from MD simulations of (TLR4)_2_ alone and (TLR4)_2_-(RipA)_2_ complex. RipA binding to (TLR4)_2_ reduced overall atomic fluctuations more pronounced at the C-terminal end indicated through the length of the associated arrows. **(K)** Pictorial representation of domain organization and positions of LC3 interacting motif regions (LIRs) present in RipA indicated by an arrowhead. CC, coiled-coil domain; SP, signal peptide. **(L)** Pictorial representation of the amphipathic nature of RipA mitochondrial targeting sequence. Colors depicting the nature of amino acids are shown.

Further, to show its specific interaction with TLR4, we used homology modeling, docking, and MD simulation studies. The Ramachandran plot, ProSA, and PROCHECK analysis of modeled and simulated RipA confirmed its stable architecture for further protein–protein modeling studies (47–49) (Figures 4A–C). Protein–protein docking results showed that the C-terminal globular domain of RipA monomers snugs into the ligand-binding pocket of TLR4 dimer. The top-scoring model from ClusPro docking of (TLR4)_2_-(RipA)_2_ heterotetramer was used as the initial structure for MD simulations (Figures 4D–G). The stability of TLR4 was assessed in terms of backbone RMSD and gyration radius (Rg), which indicates changes in fold architecture of protein during the simulation period. MD simulations stabilized its backbone RMSD and Rg (Figures 4B,C) as evident by the extensive hydrogen bond networks between 2 proteins (Figures 4H–J). Assuming that the native (TLR4)_2_ dimer architecture in the presence of its co-receptor MD2 and LPS (PDB id: 3vq2) is the active form which regulates downstream signaling, we analyzed structural integrity of the RipA bound dimer (50). The stability was assessed by constructing free energy landscapes (FEL) projected onto backbone RMSD *vs*. Rg followed by the PCA of Cα fluctuations averaged over the whole trajectory (Figures 4I,J).

In simulations of (TLR4)_2_ alone, the FEL projections showed broad distributions with 2 dominant low free energy basins corresponding to metastable states achieved by dimer. The states were characterized by high RMSD (~0.8 nm) and low Rg (~4.1–4.15 nm) compared to starting (TLR4)_2_ structure (~4.25 nm). However, in the presence of RipA, the FEL showed free energy basins corresponding to structures with low RMSD (~0.4 nm) and Rg values (~4.25–4.27 nm) comparable to starting (TLR4)_2_ architecture. These variations were identically reflected in the porcupine analysis of both systems (Figure 4J). Correspondingly, minimal Cα fluctuations were observed in RipA bound dimer, while these were substantially higher in (TLR4)_2_ alone, suggesting the overall stabilization effect of RipA. This stabilization could be attributed to a persistent H-bond network within the ligand-binding pocket of (TLR4)_2_ during the entire simulation period (Figure 4H; Supplementary Table 1). These results suggest that RipA interacts with the ligand-binding pocket of surface innate immune receptor TLR4 and likely involved in its increased expression and membrane organization.

### RipA Inhibits Cellular Autophagy in RAW264.7 Cells

**Figure 5:**
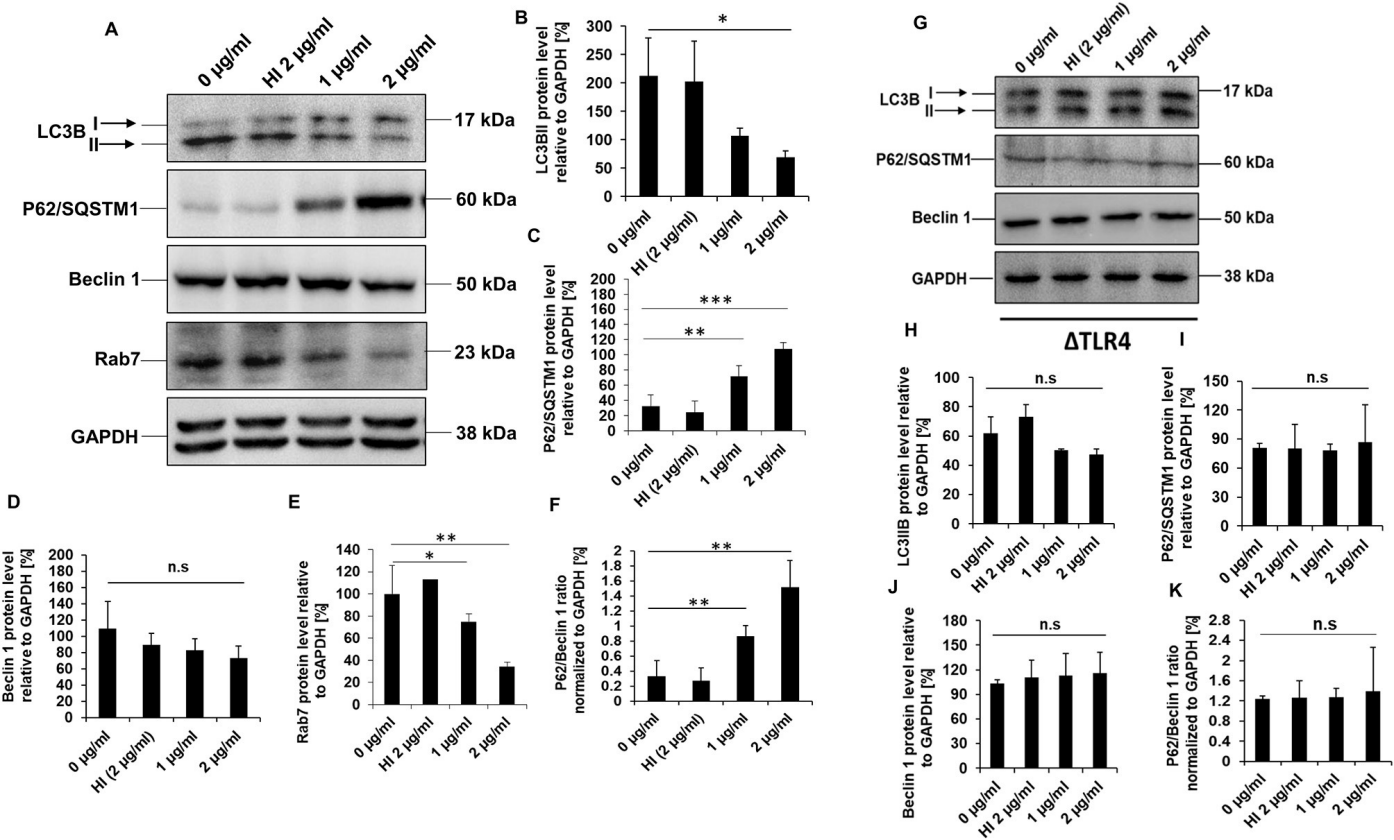
RipA inhibits autophagy in RAW264.7 cells in a TLR4 dependent manner. **(A)** Western blots demonstrating the inhibitory effect of RipA on cellular autophagy of RAW264.7 cells using autophagy markers. RAW264.7 cells were treated with RipA (1 and 2 μg/ml) for 24 h. Samples were prepared, run in SDS-PAGE, proteins were transferred onto polyvinylidene difluoride (PVDF) membrane and probed with indicated antibodies. The size of the protein bands is indicated. **(B–F)** Densitometric quantification of the protein bands (LC3BII, Beclin 1, Rab7, P62, and the ratio of P62/Beclin 1) are shown. Protein levels were represented as [%] to GAPDH. **(G)** Western blots showing the levels of autophagy markers in TLR4 knockout cells. Untreated and HI treated cells were used as negative controls. GAPDH was used as a loading control. **(H–K)** Densitometric quantitation of LC3BII, P62, Beclin1, and the ratio of P62/Beclin1 in treated ΔTLR4 cells. Protein levels of autophagy markers in ΔTLR4 cells were expressed as [%] to GAPDH. n.s indicates not significant. Data are representative of 3 independent experiments and expressed as means ± SD. *p < 0.05, **p < 0.01, and ***p < 0.001 vs. controls. n.s, not significant.

*In-silico* analysis of RipA protein sequence revealed the presence of LC3 interacting motifs, which are known to play an essential role in autophagy regulation (Figure 4K). Thus, we further proceeded to understand the possible mechanistic details by which RipA subverts host cell functions to modulate the cellular autophagy pathway. RAW264.7 macrophage cells were treated with RipA, and cellular autophagy activity was analyzed using Western blot by measuring 4 classical parameters: the conversion of LC3BI to LC3BII along with the levels of autophagy substrate P62/SQSTM1, Beclin 1, and Rab7 (Figures 5A–E). RipA treatment significantly decreased LC3BII and Rab7 levels and caused the accumulation of autophagy substrate P62. In contrast, no significant change in Beclin 1 levels was observed, indicating that cellular autophagy activity is repressed in the presence of RipA (Figures 5B–E). To further strengthen this finding, we calculated the P62/Beclin 1 ratio and observed an increase in the ratio in a dose-dependent manner (Figure 5F). Next, we checked whether the inhibitory effect of RipA on cellular autophagy is also dependent on TLR4. The Western blot analysis of RipA-treated lysates from the ΔTLR4 cells to measure the cellular levels of LC3BII, P62, Beclin 1, and P62/Beclin 1 ratio revealed no significant changes in the levels of these autophagy markers in ΔTLR4 cells (Figures 5G–K). These results demonstrate that RipA inhibits autophagy mediated by innate immune receptor TLR4.

### RipA Abolishes the Autophagy Inducing Effect of Rapamycin and Potentiates Bafilomycin A1

**Figure 6:**
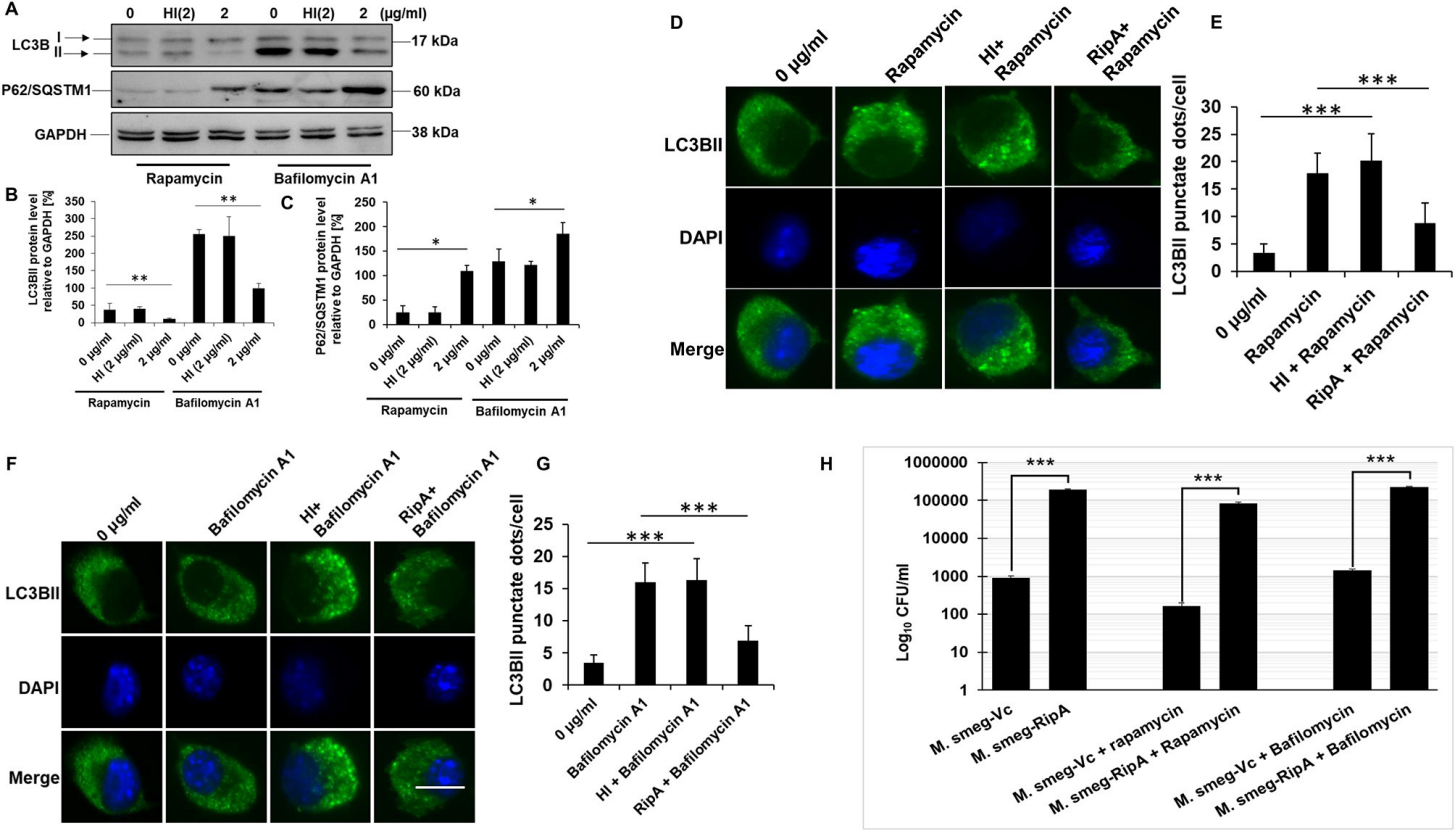
RipA inhibits autophagy initiation in RAW264.7 cells. **(A)** Western blots showing the levels of autophagy markers LC3BII and P62 in RAW264.7 cells treated with RipA in the presence of rapamycin (200 nM) and bafilomycin A1 (50 nM). Untreated and HI treated cells were used as negative controls. GAPDH was used as loading control. **(B,C)** Densitometric quantifications of LC3BII and P62 bands are shown relative to GAPDH [%] as bar graphs. **(D)** Immunofluorescence microscopic images demonstrating the LC3 foci in untreated, Rapamycin (200 nM) treated, HI + Rapamycin treated, and RipA + Rapamycin treated RAW264.7 cells. DAPI was used to stain the nucleus. Untreated and rapamycin-treated cells were used as negative and positive controls. **(E)** The average number of LC3 foci were counted and represented as a bar graph. **(F)** Immunofluorescence microscopic images showing the LC3 foci in untreated, HI treated, and RipA-treated RAW264.7 cells in the presence of bafilomycin A1 (50 nM). Untreated and bafilomycin A1 treated cells were used as negative and positive controls. **(G)** The average number of LC3 foci were counted and represented as a bar graph. **(H)** RAW264.7 cells were infected with recombinant *M. smegmatis* containing RipA or vector alone harboring plasmids (MOI-1:10) for 24 h in the presence of rapamycin (200 nm) and bafilomycin A1 (50 nm) or without drug. Colony-forming unit (CFU) of vector alone and RipA transformed recombinant *M. smegmatis* were determined after 24 h of infection and represented as Log_10_ CFU/ml. Data are representative of 3 independent experiments and expressed as means ± SD. *p < 0.05, **p < 0.01 and***p < 0.001 vs. controls.

To understand the molecular mechanism of how RipA impairs autophagy, we analyzed the cellular autophagy level in RAW264.7 cells in the presence of rapamycin and bafilomycin A1. Rapamycin is an inhibitor of autophagy master regulator mTORC1 kinase; therefore, the treatment of macrophage cells with rapamycin will induce autophagy. RipA, being an inhibitor of autophagy, is expected to mitigate the effect of rapamycin. Bafilomycin A1 is an inhibitor of autophagy which inhibits the fusion of autophagosomes to lysosomes at the late stage of autophagy and is used to study autophagic flux. Treatment with bafilomycin A1 leads to an increased expression of LC3BII due to inhibition of its degradation. The RipA treatment of macrophage cells also reduces the autophagic flux that could be visualized by reduction in the effect of bafilomycin A1. We treated macrophage cells with RipA (2 μg/ml) in the presence of rapamycin (200 nM) or bafilomycin A1 (50 nM) followed by Western blot analysis. Conversion of LC3BI to LC3BII and utilization of autophagy substrate P62 were measured. RipA significantly inhibited autophagy in the presence of rapamycin and bafilomycin A1, as indicated by the lower conversion of LC3BI to LC3BII and decreased consumption of substrate P62 (Figures 6A–C). To further confirm the inhibitory effect of RipA on cellular autophagy, we employed immunofluorescence microscopic analysis and measured the LC3BII puncta formation in the presence of these pharmacological agents. Significantly reduced numbers of punctate foci, the indicator of autophagic membranes, were observed in RipA treated samples. The number of punctate foci were counted in various fields and represented as bar graphs (Figures 6D–G). These results suggest that RipA inhibited the cellular autophagy initiation in RAW264.7 cells by repressing the formation of LC3BII associated membrane structure.

*Mycobacterium smegmatis* is a very commonly used model organism to understand the functional attributes of *M. tb* genes which are essential and therefore cannot be knocked out (51–53). *ripA* is an essential gene of *M. tb*; therefore, we employed recombinant *M. smegmatis* strain expressing RipA to validate our *in-vitro* observations (54). *M. smegmatis* infection of macrophage cells induces robust activation of the autophagy pathway which mediates its clearance (55). Our observation (Figures 5, 6) that RipA inhibits cellular autophagy in RAW264.7 cells prompted us to investigate these effects in *M. smegmatis*, a surrogate model of *M. tb*. Though *M. smegmatis* also contains RipA; however, it was interesting to observe that it does not harbor the highly conserved canonical LC3 interacting region (LIR) motif, suggestive of its inherent incapability to modulate host autophagy machinery. On the contrary, *M. tb* RipA has a high binding affinity LIR motif that may enable it to regulate the autophagy mediated innate defense (Supplementary Figure 3). Recombinant *M. smegmatis* constitutively expressing RipA was generated by cloning into shuttle vector pST-2K (32) to study its function in native condition. The RipA expression was confirmed by the Western blot analysis using anti-RipA antibody (Supplementary Figure 2E). Though *M. smegmatis* also encodes RipA, the sequence identity with *M. tb* homolog was found to be <65%, which possibly led to the functional divergence of the pathogenic protein. All the results implicated *M. tb* RipA in the observed effects with no role of endogenous RipA expression in *M. smegmatis*. Infection of macrophage cells with recombinant *M. smegmatis* expressing *M. tb* RipA showed enhanced intracellular survival (Figure 6H), whereas wild-type *M. smegmatis* and *M. smegmatis* transformed with vector control were cleared at similar rate. We further analyzed the survival of recombinant *M. smegmatis* in macrophage cells by analyzing the colony-forming unit (CFU) in the absence or presence of rapamycin and bafilomycin A1. RipA increased the survival of *M. smegmatis* in macrophages, and the treatment of rapamycin reduces the CFU. On the other hand, bafilomycin A1 treatment increased the survival supporting our Western blot results (Figure 6H). The decreased LC3BI lipidation and P62 utilization suggest that the virulence factor RipA may attenuate the early step of autophagy initiation by activating the signaling cascade that is involved in autophagy repression. These results suggest that RipA interrupts the initiation of cellular autophagy in RAW264.7 cells.

### RipA Activates PI3K-AKT-mTORC1 Signaling Axis Mediated by TLR4 Activation

**Figure 7:**
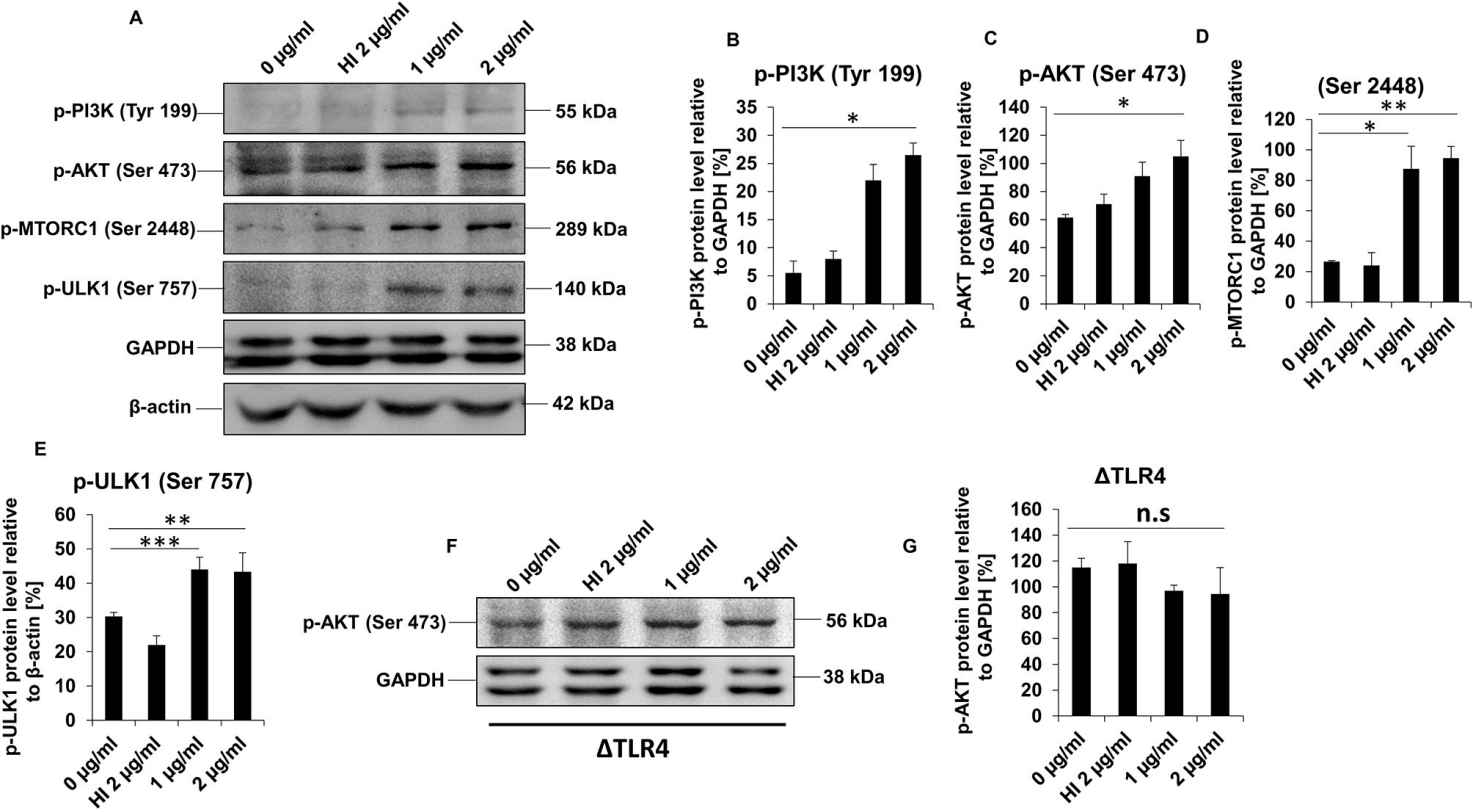
RipA activates PI3K-AKT-mTORC1 signaling cascade mediated by TLR4. **(A)** Western blots showing levels of phospho PI3K, p-AKT, p-mTORC1, and p-ULK1 kinases in RipA-treated RAW264.7 cells. GAPDH was used as a loading control. **(B–E)** Quantitation of the p-PI3K, p-AKT, p-mTORC1, and p-ULK1 protein bands are shown as bar graphs. Protein bands are normalized to GAPDH or β-actin and represented as [%] protein level to GAPDH or β-actin. **(F)** Western blots showing the expression level of phospho AKT in RipA-treated ΔTLR4 cells. Untreated and HI treated cells were used as negative controls. **(G)** Densitometric analysis of phospho AKT bands in ΔTLR4 cells is shown as a bar graph. Phospho AKT levels were expressed as relative protein levels to GAPDH [%]. Data are representative of 2 independent experiments and expressed as means ± SD. *p < 0.05, **p < 0.01, and ***p < 0.001 vs. controls. n.s, not significant.

Previous studies have established that an active PI3K-AKT-mTORC1 signaling cascade has an inhibitory effect on autophagy (56). Given the fact that RipA inhibits the initiation of autophagy, we hypothesized that the virulence factor RipA might activate the PI3K-AKT-mTORC1 signaling pathway for the inhibition of autophagy initiation. We analyzed the activation and repression status of PI3K-AKT-mTORC1 and ULK1 by detecting activator and inhibitory phosphorylation using phospho-specific antibodies in the Western blot analysis. We observed that treatment with RipA consistently stimulated activation of PI3K-AKT-mTORC1 and inhibition of ULK1 protein kinases in a dose-dependent manner, as evident from phosphorylation status. The phospho-activation of PI3K-AKT-mTORC1 induces the phosphorylation of ULK1 culminating in the repression of autophagy, which is indicated by the dose-dependent increase in its inhibitory phosphorylation (Figures 7A–E). These results suggest that RipA induced the activatory phosphorylation of PI3K-AKT-mTORC1 and inhibitory phosphorylation of autophagy kinase ULK1. Reduced activation of PI3K-AKT-mTORC1 and minimal inhibition of ULK1 were observed when denatured protein (HI) was incubated with cells. However, the treatment of RipA to ΔTLR4 cells did not show any change in the activation status of AKT, one of the kinases of the pro-survival pathway (Figures 7F,G). Therefore, we conclude that RipA activates TLR4-dependent PI3K-AKT-mTORC1 signaling pathway involved in the inhibition of autophagy initiation.

### RipA Localizes to the Mitochondria and Inhibits the Production of Electron Transport Chain Proteins in Macrophages

**Figure 8:**
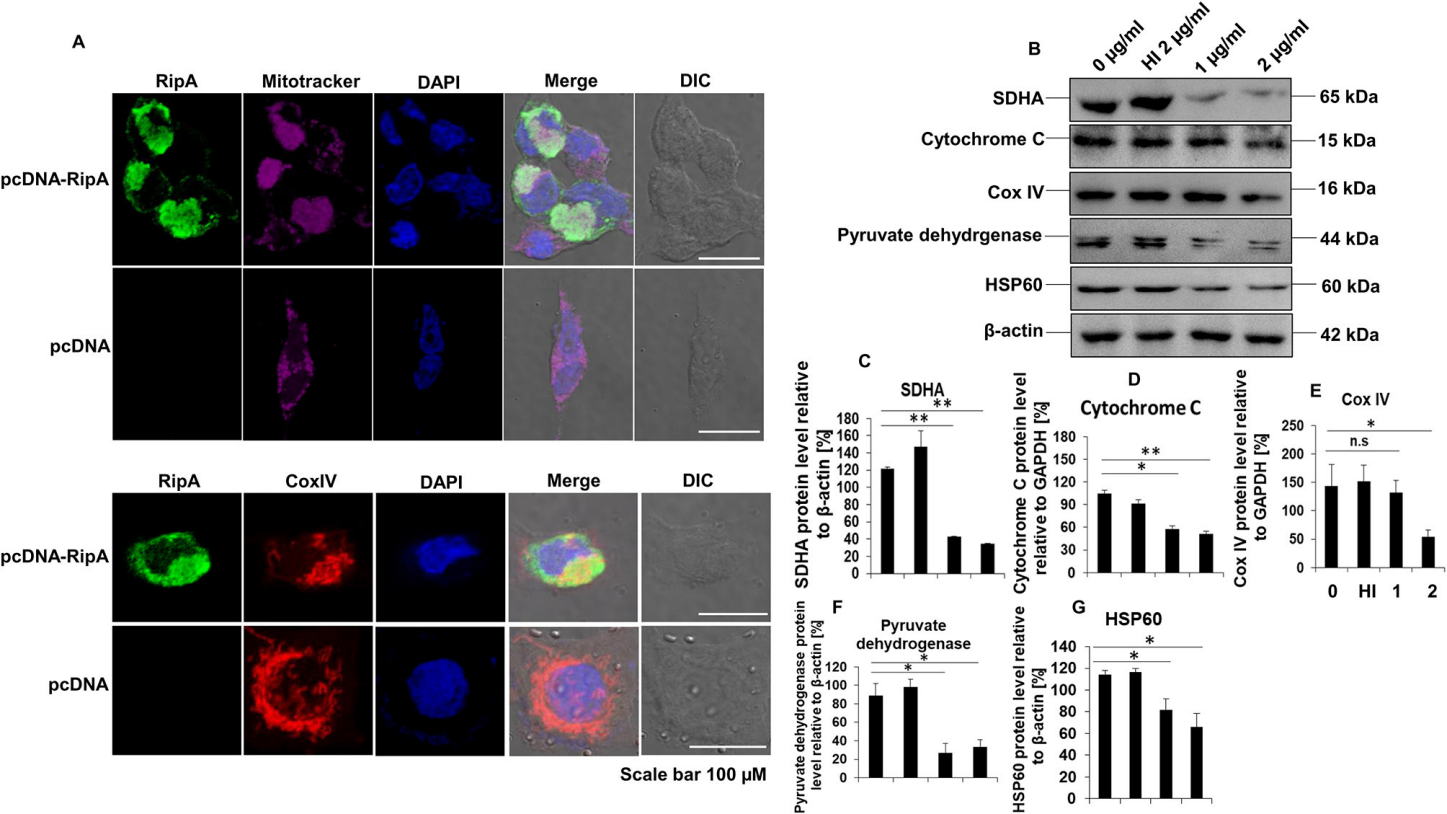
RipA localizes to the mitochondria and treatment of RipA to RAW264.7 cells lead to decreased protein levels of electron transport chain enzymes. **(A)** Confocal microscopic images showing mitochondrial co-localization of untagged RipA at 48 h after transfection. Colocalization of untagged RipA (probed with anti-RipA antibody, 1:500) with mitochondria stained with MitoTracker Deep Red FM dye and CoxIV. Vector alone transfected cells were used as the negative control. Scale bar indicates 100 μm. Anti-RipA antibody was used to probe the localization of RipA. Mitochondrial positions were marked using MitoTracker Deep Red FM dye and CoxIV protein of mitochondrial electron transport chain. DAPI was used to stain the nucleus. A488 and A594 conjugated secondary antibodies were used for signal detection. **(B)** Western blots showing the levels of electron transport chain enzymes in RipA treated RAW264.7 cells. Untreated and HI treated cells were used as negative controls. **(C–G)** Densitometric quantification of SDHA, cytochrome C, CoxIV, pyruvate dehydrogenase, and HSP60 are shown as bar graphs. Data are representative of 2 independent experiments and expressed as means ± SD. n.s, not significant. *p < 0.05 and **p < 0.01 vs. controls.

RipA interacts with mammalian cell entry protein Mce2B (29), suggesting that RipA is targeted to the host macrophage cells to modulate host-pathogen interaction. Thus, to explore its localization inside the mammalian cells, we employed transient transfection of C-terminal GFP-tagged RipA into HEK293T cells and analyzed its localization using fluorescence microscopy. GFP-tagged RipA localized into the cytoplasm as punctuated foci, indicating that it might be targeted to a specific organelle (Supplementary Figure 4). On the contrary, the vector alone transfected cells displayed diffuse signal all across the cytoplasm. *In-silico* analysis by various localization servers like MITOPROT, PSORTII, and TargetP predicted the presence of N-terminal mitochondrial targeting sequence in RipA and possible mitochondrial localization with high probability. Mitochondrial targeting sequences are amphipathic, and upon analysis, we observed that half of the RipA mitochondrial targeting sequence is formed by the predominantly positively charged amino acids followed by hydrophobic amino acids demonstrating its amphipathic nature (Figure 4L). We used untagged RipA to study its co-localization to the mitochondria assessed using MitoTracker Deep Red FM dye and CoxIV. After transient transfection of untagged RipA or pcDNA 3.1^+^ vector alone, we observed that RipA is co-localized along with mitochondria (Figure 8A).

To confirm this observation, untagged RipA cloned in pc-DNA3.1^+^ vector or vector alone were transfected in HEK293T cells, and the location of RipA was studied at 24, 48, and 72 h post-transfection using a polyclonal anti-RipA antibody. Analysis of immunofluorescence images showed that RipA co-localized along with MitoTracker Deep Red stained mitochondria (Supplementary Figure 5A). Statistical quantitation of RipA and MitoTracker Deep Red stained mitochondria is shown in Supplementary Figure 5B. Colocalization of untagged RipA to the mitochondria suggest that RipA is a mitochondria-targeted protein. Vector alone transfected cells determined the specificity of the anti-RipA antibody by depicting the regular distribution of mitochondria in the cytoplasm (Figure 8A). This observation prompted us to explore the role of RipA in regulating mitochondrial function. We examined the levels of oxidative phosphorylation enzymes, such as succinate dehydrogenase (SDHA) cytochrome C, CoxIV, and pyruvate dehydrogenase, as well as HSP60 in RipA treated cells. As shown in Figures 8B–G, treatment with RipA resulted in lower levels of SDHA, cytochrome C, CoxIV, and pyruvate dehydrogenase of the mitochondrial oxidative phosphorylation as well as chaperone HSP60. These results demonstrate that RipA localizes to the mitochondria and possibly hampers normal mitochondrial function.

### RipA Inhibits Caspase-Dependent Apoptosis Through the Activation of MDM2 and the Map Kinase JNK 1/2

**Figure 9:**
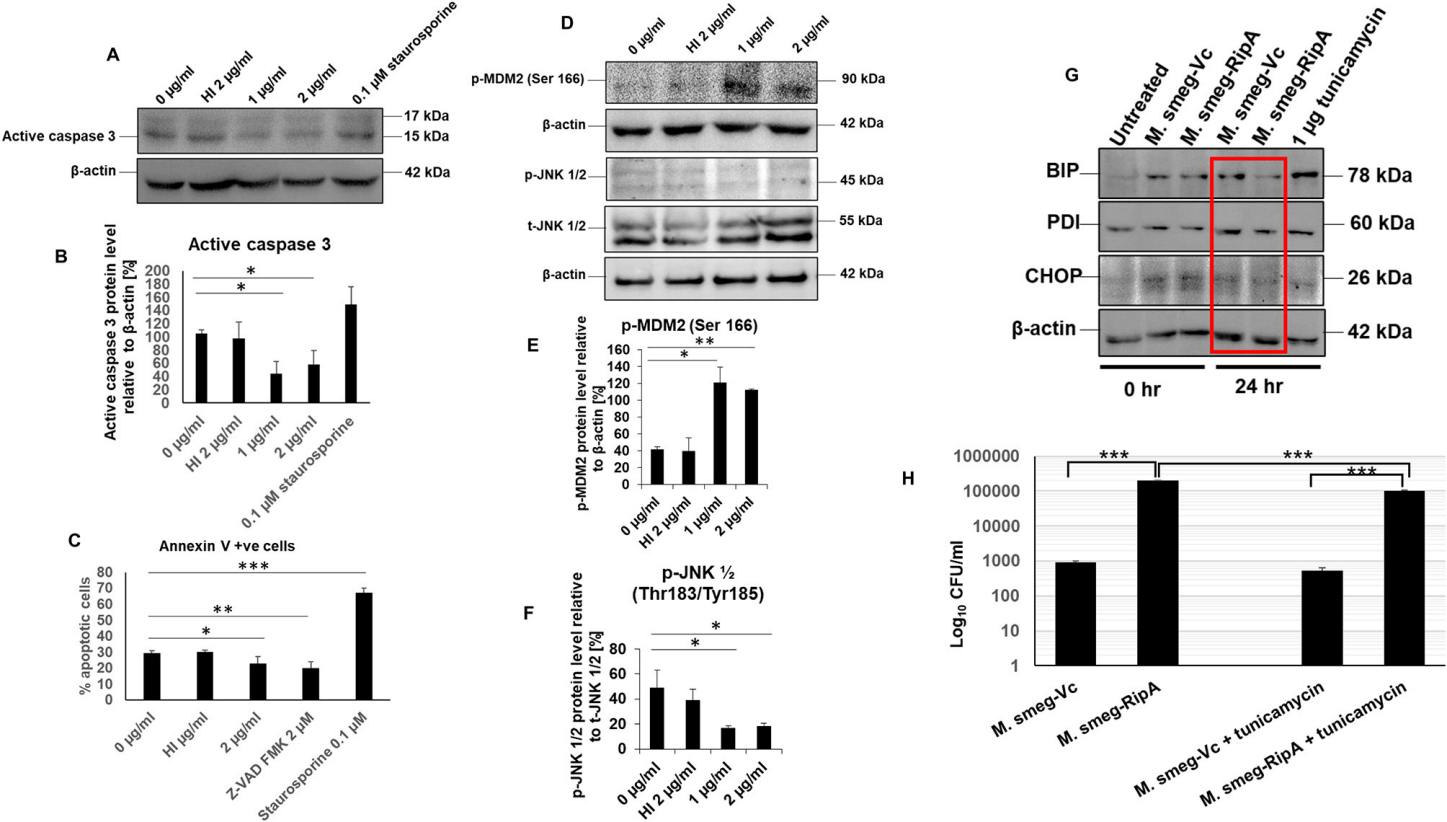
RipA inhibits caspase-dependent apoptosis of RAW264.7 cells. **(A)** Western blots showing the level of active caspase 3 in RipA treated samples. Untreated and HI treated cells were used as negative controls. **(B)** Densitometric quantitation of active caspase 3 in RipA treated samples. **(C)** Flow cytometric analysis of early apoptotic cells in RipA treated macrophages. HI-treated cells were used as a negative control, whereas staurosporine and ZVAD-FMK served as controls for caspase-dependent apoptosis induction and repression. **(D)** Western blots showing the level of activated phospho MDM2 and inhibited phospho JNK 1/2, and total JNK 1/2. Total JNK 1/2 and β-actin were used as controls. **(E,F)** Densitometric quantification of pMDM2 and pJNK 1/2 protein bands are shown as bar graphs. **(G)**. Western blots showing the levels of ER stress markers BIP, PDI, and CHOP. **(H)** RAW264.7 macrophage cells (2 × 10^6^ cells) were infected with recombinant *M. smegmatis* expressing *M. tb* RipA and vector alone at MOI-1:10. Colony-forming units were determined after 24 h of infection. Recombinant *M. smegmatis* containing RipA showed higher survival inside macrophages in untreated, as well as tunicamycin treated cells demonstrating higher infectivity and virulence capacity of recombinant *M. smegmatis* containing RipA. Data are representative of 3 independent experiments and expressed as means±SD. *p < 0.05, **p < 0.01, and ***p < 0.001 vs. controls.

We have shown in previous results that RipA activates PI3K-AKT-mTORC1 pro-survival signaling cascade of the host that results in the inhibition of programmed cell death/apoptosis (57). It prompted us to explore the role of RipA in regulating apoptosis by determining the activation status of caspase 3, AKT downstream target MDM2, Map kinase JNK 1/2, and surface exposure of phosphatidylserine. RAW264.7 cells were treated with RipA alone (1 or 2 μg/ml) or with Z-VAD-FMK and staurosporine. After 24 h of incubation, the cells were stained with Annexin V/PI, or the Western blot analysis was performed. RipA treatment significantly reduced the executioner caspase 3 cleavage (Figures 9A,B). RipA also decreased Annexin V or Annexin V+PI double-positive cells (Figure 9C and Supplementary Figure 6). RipA treatment of macrophage cells resulted in an enhanced activation of AKT downstream target MDM2, which is a regulator of the P53 dependent apoptotic pathway as well as inhibition of MAP kinase JNK 1/2. β-actin and total JNK 1/2 were used as loading controls in Western blot analysis. Phosphorylated MDM2 and JNK 1/2 were normalized to respective β-actin and total JNK 1/2 and represented as bar graphs. As shown in (Figures 9D–F), RipA induced the phosphorylation and activation of MDM2, whereas it inhibited the phosphorylation of MAP kinase JNK 1/2, which is suggestive of its inhibitory role. These results demonstrate the anti-apoptotic potential of RipA on macrophage cells, which can be exploited by the pathogen for its survival and dissemination.

### RipA Modulates Endoplasmic Reticulum Stress Response by Inhibiting Unfolded Protein Response Pathway

The role of RipA in metabolic reprogramming and inhibition of apoptosis led us to explore its role in the unfolded protein response (UPR) pathway that is indicative of ER stress. Macrophages were infected with recombinant *M. smegmatis* bacteria expressing RipA constitutively to study the modulation of the ER stress response. Downregulation of CHOP in recombinant bacteria-infected macrophages suggested ER stress mitigation by RipA (Figure 9G). Consequent to it, the levels of the UPR pathway proteins, BIP and PDI, were analyzed, which also depicted the downregulation after 24 h of infection (Figure 9G). Our earlier observation that RipA inhibited apoptosis support these findings. The infection of macrophage cells with *M. smegmatis* induces robust activation of the UPR mediated apoptosis pathway for its clearance. Inhibition of UPR markers by recombinant *M. smegmatis* containing RipA suggests that RipA inhibits the activation of ER-mediated UPR response.

Further, to confirm the role of UPR in cell survival, we used tunicamycin as an agent to induce the general UPR pathway of ER. Tunicamycin inhibits glycosylation into the ER lumen. Macrophage cells infected with recombinant *M. smegmatis* or vector transformed were treated with tunicamycin, and bacterial survival was measured in terms of CFU. Tunicamycin treatment leads to the killing of intracellular *M. smegmatis* (wild-type and vector transformed *M. smegmatis*); however, the killing was markedly reduced in the case of recombinant *M. smegmatis* (Figure 9H). The higher CFU in tunicamycin treated recombinant *M. smegmatis* suggested that RipA inhibits tunicamycin induced UPR. These observations confirm the role of RipA in stress reduction, which is evidenced by a lower expressions of the UPR pathway chaperones, BIP and PDI, as well as a reduced expression of CHOP. These findings suggest that the inhibition of the UPR response is yet another role of RipA in cell survival response.

## Discussion

We demonstrate that an *M. tb* secretory protein RipA activates macrophages to produce pro-inflammatory cytokines, TNF-α, IL-12, and IL-6, *via* the activation of TLR4. These results, along with the enhanced expression of co-stimulatory and antigen-presenting molecules, demonstrate that RipA likely stimulates the development of Th1 immune response *via* macrophage M1 polarization. Our results also demonstrate that RipA stimulates the overexpression of TLR4 and enhances its membrane organization, which is a hallmark feature of TLR4 activation. MD simulations exhibited that RipA acts as a stabilizing agonist of (TLR4)_2_ dimer. This pro-inflammatory response *via* TLR4 activation could be reminiscent of the canonical host immune strategy to arm the immune system against pathogens by exploiting antigenic regions of virulence factors (58). However, pathogens can also exploit the enhanced inflammatory response to induce host immunopathology for their dissemination (59, 60). Increased TNFα levels account for unwanted inflammatory effects and an unusual clinical deterioration (61). Furthermore, recent advances in tuberculosis (TB) immunity have revealed that granulomatous inflammation in TB infection is highly dynamic and the early influx of neutrophils may lead to excessive inflammation and pulmonary cavitation, which provide niches for *M. tb* not only to survive but also to spread to other sites. It is worthwhile to note that, even though the host can evoke pro-inflammatory responses against a protein, these virulence factors deceive the host by modulating the host defense mechanism for their survival.

Autophagy and apoptosis are two critical innate defense mechanisms against invading intracellular bacterial pathogens. These two innate defense pathways are linked both positively and negatively, and extensive crosstalk exists between the two processes. Generally, the activation of autophagy blocks the induction of apoptosis, whereas apoptosis-associated caspase activation shuts off the autophagic process (62, 63). However, during intracellular infection, host cells could activate both autophagy and apoptosis for the clearance of the pathogen. RipA inhibits autophagy so that host cells are not able to activate autophagy adaptors to induce xenophagy. Moreover, due to autophagy inhibition, the host cell is not able to adapt to the pathogen mediated stress that aids in pathogen survival. Intriguingly, RipA also ensures the inhibition of apoptosis that acts as a canonical innate defense strategy to inhibit the intracellular survival of the pathogen. In the present study, we used a combination of *in-silico* analyses, *in-vitro* mammalian cell transfection experiments, and recombinant *M. smegmatis*, a surrogate of *M. tb*, to describe a previously uncharacterized mechanism of autophagy blockade by the *M. tb* secretory effector protein, RipA. The use of rapamycin and bafilomycin A1 identified that RipA acts as an inhibitor of autophagy initiation as it reduced the effect of rapamycin as well as decreased autophagic flux that diminished the effect of bafilomycin A1. This autophagy inhibition involves the TLR4 mediated activation of the PI3K-AKT-mTORC1 signaling pathway (64). Interestingly, we also observed that RipA treated RAW264.7 cells exhibited a low level of Rab7, a member of small GTPases. Rab7 is one of the most important molecules that modulate the maturation of autophagosomes and is an effective multifunctional regulator of autophagy (65). These findings possibly indicate that RipA inhibits autophagy initiation through the activation of PI3K-AKT-mTORC1 signaling axis and autophagosomes maturation by inhibiting the production of Rab7. The innate immune receptor TLR4 has also been known to mediate LPS-induced autophagy (66), but similar to our observation, it also mediated the inhibition of autophagy in various cases (67, 68). The activation of receptor along with downstream signaling and properties of the ligand ultimately translates into the desired effect. LPS activates TLR-mediated autophagy that is TRIF dependent and Myd88 independent. We observed that RipA-induced the inhibition of autophagy by TLR4 activation that is Myd88 dependent.

RipA stimulated macrophages also displayed the inhibition of caspase-mediated apoptosis *via* the suppression of active caspase 3 and MAP kinase JNK 1/2 along with the activation of AKT downstream target ubiquitin ligase MDM2. This observed an inhibitory effect of RipA on apoptosis, which may likely be through the activation of MDM2 by AKT. These findings suggest that *M. tb* RipA dampens two of the crucial host-mediated defense pathways, autophagy and apoptosis, to favor the survival of pathogen inside the infected host (69). Several of the *M. tb* proteins are known to inhibit/induce host cell apoptosis or autophagy. PE_PGRS41 is the only other known protein that has been demonstrated to inhibit apoptosis as well as autophagy (70). NuoG, a type I NADH dehydrogenase of *M. tb*, inhibits extrinsic apoptosis and autophagy, whereas ESAT-6 is involved in the inhibition of autophagy and induction of apoptosis (23, 24, 71). Eis, another protein of *M. tb*, is involved in suppressing autophagy in a redox-dependent manner and also inhibits caspase-independent cell death (22).

Our observation that RipA inhibits the production of UPR pathway chaperones, BIP, and PDI, as well as CHOP, suggest that RipA represses the ER-mediated UPR pathway to inhibit apoptosis for increased bacterial survival. ER-mediated stress response or activation of the UPR pathway plays a significant role in reducing the survival of *M. tb* and in suppressing apoptosis. We observed that RipA increased the survival of recombinant *M. smegmatis* expressing RipA within the macrophages. The recombinant bacteria also exhibited enhanced resistance to ER stress inducer tunicamycin, thereby reiterating the role of RipA in repressing apoptosis and causing increased survival within macrophages.

Reduced levels of oxidative phosphorylation enzymes SDHA, cytochrome C, and CoxIV in RipA-treated macrophages suggest that *M. tb* RipA promotes Warburg-like effect or metabolic repurposing. It may provide the *M. tb*, a favorable niche for replication, possibly through rewiring TCA cycle intermediates, such as citrate and succinate for biosynthetic pathways of the pathogen. It has been earlier demonstrated that *M. tb* provokes Warburg-like metabolic effects by inhibiting oxidative phosphorylation and inducing aerobic glycolysis (72, 73). However, the role of individual effector proteins of *M. tb* has not yet been elucidated. *M. tb* inevitably uses a battery of virulence factors like RipA to target the cellular physiology to perturb the immunometabolic machinery. Mitochondria being the power house of cells are target of intracellular pathogens like *M. tb* that ensures rewiring of host metabolites for better intracellular survival. The downregulation of electron transport chain and Tricarboxylic acid cycle enzymes by RipA marks an energy quiescent cell that is permissive to mycobacterial infections. The Warburg-like effect induced by RipA translates into increased glucose uptake and glycolysis along with the deviation of glycolytic intermediates to fatty acids, which acts as a source of nutrition for intracellular mycobacteria. The classical foamy phenotype of infected macrophages is due to rewiring of the glycolytic pathway toward ketone body and lipid synthesis. Infected macrophages switch from pyruvate oxidation to the reduction of pyruvate into lactate that serves as an additional carbon substrate for *M. tb* (74–76). Although, yet to be validated, these effects point to TLR4-mediated HIF1-α activation possibly lead to the inhibition of oxidative phosphorylation and the activation of glycolysis to promote metabolic reprogramming. RipA is also localized to mitochondria where it can likely impair cellular bioenergetics. Further definitive studies are warranted to delineate the role of RipA targeted to the mitochondria in the regulation of its function and metabolic reprogramming. In addition to inhibition of the production of mitochondrial oxidative phosphorylation enzymes, RipA also inhibited the production of stress-activated mitochondrial chaperone HSP60, which plays an unequivocal role in the mitochondrial protein import and quality control along with mitigation of host stress (77).

**Figure 10:**
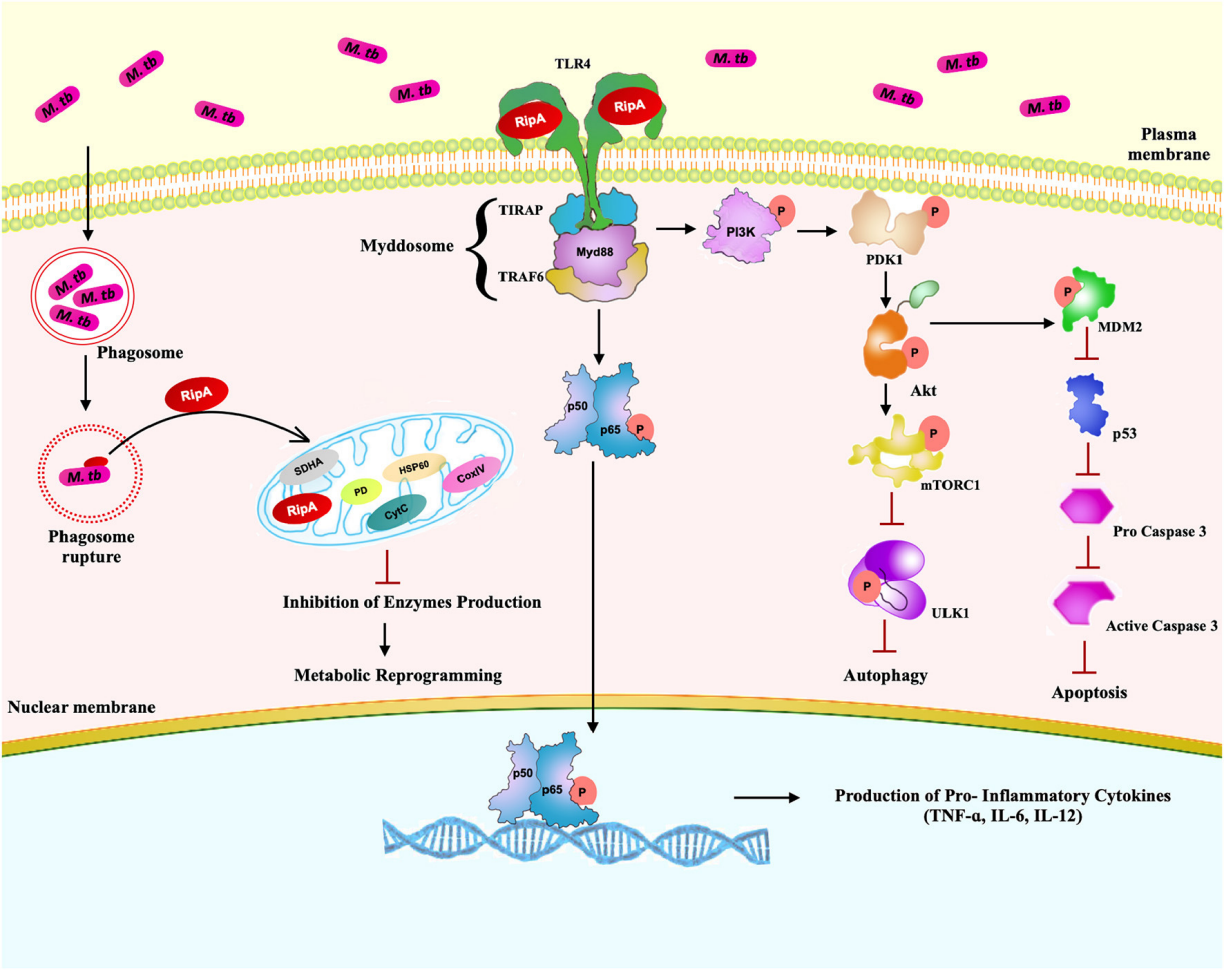
A schematic model of RipA depicting possible interaction of RipA with surface immune receptor TLR4 and emanating downstream cascades. After interaction with TLR4, RipA activates a downstream PI3K-AKT-mTORC1 signaling pathway that ultimately culminates in the repression of autophagy and apoptosis. RipA recognition by TLR4 activates canonical NFκB signaling pathways and induces the production of pro-inflammatory cytokines. RipA also inhibits the production of oxidative phosphorylation enzymes resulting in metabolic reprogramming.

Summarizing these observations, we propose a model (Figure 10) in which *M. tb* RipA interacts with the surface immune receptor, TLR4. Interaction of RipA to TLR4 results in the activation of host pro-survival signaling cascade (PI3K-AKT-mTORC1) that, in turn, represses two of the host defense pathways, autophagy, and apoptosis. RipA also induces metabolic repurposing of macrophages and the modulation of autophagy and apoptosis that may represent a key virulence strategy used by *M. tb* through RipA to replicate within macrophages and cause successful pathology. Though the findings were interesting, these need to be validated in RipA deletion mutant of *M. tb*. The multifaceted role of this protein ought to be further correlated with the pathophysiology of the *M. tb* infection. The suggested effect on metabolic reprogramming demands validation and correlation with the suppression of cell death pathways. Further, correlation of observed effects with actual physiological concentration of RipA during the *in-vivo* infection will aid in better understanding the moonlighting effect of this protein. Multi-tasking and protein promiscuity (78, 79) is emerging as a common strategy for pathogens that have evolved by genomic reduction (80, 81). A recent report also showed the role of RipA in persistence of *M. tb* in mice suggestive of its role as a virulence factor and potential drug target (82). This corroborates our observation of the pro-pathogen role of RipA, wherein we speculate that this virulence factor possibly leads a multipronged attack to subdue host defense. While one of the emphasis of this work revolves around autophagy, the other substantial moonlighting functions, like disruption in cellular bioenergetics and immune modulation, lead to a multipronged attack to dampen host response point to RipA as a crucial virulence factor. Being an endopeptidase, it catalyzes a crucial step in cell wall synthesis that is one of the major virulence determinants of *M. tb*. Moreover, RipA is critical for virulence as *M. tb* ΔRipA failed to establish the infection in macrophages as well as the mouse model of TB (82). This is an avid example of functional fluidity in pathogenic proteins that we believe *M. tb* has gained after reductive evolution. Thus, we firmly believe that RipA is a potential drug target and further studies on this protein can provide insights into pathomechanisms employed by chronic pathogens like *M. tb*. Further studies in this area could delineate the RipA mediated pathology for better understanding of disease pathomechanism apart from a potential cell wall targeting drug against *M. tb*.

## Data Availability Statement

The original contributions presented in the study are included in the article/Supplementary Material, further inquiries can be directed to the corresponding author/s.

## Author Contributions

NE and SH: conceptualize the project, funding acquisition, investigation, project administration, and supervision. MS, NQ, NS, JS, JAS, MK, NE, and SH: data curation and analysis. MS, NE, and SH: writing manuscript draft. MS, JAS, NQ, NE, and SH: manuscript review and editing. All authors contributed to the article and approved the submitted version.

## Conflict of Interest

The authors declare that the research was conducted in the absence of any commercial or financial relationships that could be construed as a potential conflict of interest.

## Supplementary Material

Supplementary Figure 1 Analysis of antigenic potential and B cell epitopes of RipA. **(A)** The antigenic propensity of RipA was analyzed using the Scratch protein predictor tool and compared with known antigenic molecules of *Mycobacterium tuberculosis* (*M. tb*) such as ESAT-6, CFP-10, and PSTS1. As shown in the pictorial representation, RipA displays the comparable antigenic potential to the well-known antigenic molecules of *M. tb*. **(B)** B-cell epitope prediction was performed using a B-cell epitope prediction tool. As shown in the figure, RipA harbors sequence stretches that form B-cell epitopes. B-cell epitopes present in RipA were similar to known proteins that have abundant B-cell epitopes such as ESAT-6, CFP-10, and PSTS1.

Supplementary Figure 2 Protein purification profile of RipA and its effect on cell survival of RAW264.7 cells. **(A)** SDS-PAGE profile of affinity-purified RipA. **(B)** Western blot showing the specificity of the anti-RipA antibody and purity of RipA protein. **(C)** MTT assay was used to study the effect of RipA on the survival of RAW264.7 cells. RAW264.7 cells were treated with various concentrations of RipA (1, 2, 5, and 10 μg/ml), and cell survival was measured using MTT assay after 24 h of treatment. Untreated cells were used as control. Cell survival in the presence of RipA was analyzed as compared to untreated cells and represented as [%] survival of RAW264.7 cells. **(D)** Western blot showing proteinase K digested and heat-inactivated (HI) protein samples as well as purified RipA alone probed using anti-RipA antibody. One aliquot of purified RipA was subjected to proteinase K digestion followed by heat inactivation at 100°C for 4 h. Proteinase K digested and HI sample was used as a negative control in each experiment as HI RipA. **(E)** Western blot showing the expression level of RipA in *M. smegmatis* transformed with pST-2K vector alone and pST-2K containing *ripA*. Western blot was performed using anti-RipA polyclonal sera. The enhanced expression of surface markers on THP-1 cells (MHC-II and CD80) at 24 h after treatment with medium alone, and 2 μg/ml RipA. The expression level was determined by FACS analysis using A488 and PE linked monoclonal antibodies, respectively. **(F)** Graphical representation of MHC-II. Quantitative representation of MHC-II. Graphical representation of CD80. Quantitative representation of CD80 expression on the surface of THP-1 cells. Data are representative of three independent experiments and expressed as means ± SD. ^*^*p* < 0.05 and ^**^*p* < 0.01vs. controls.

Supplementary Figure 3 *M. tb* RipA contains two canonical LIR motifs. **(A)** The RipA protein sequence of *M. tb* and *Mycobacterium smegmatis* was analyzed using iLIR web-server (www.repeat.biol.ucy.ac.cy/cgi-bin/iLIR/iLIR_cgi). *M. tb* RipA was found to encompass a high scoring LC3 interacting motif region (LIR) motif (RAWDGL) that was absent in *M. smegmatis* RipA. **(B)** Multiple Sequence Alignment of *M. tb* RipA and *M. smegmatis* RipA reveals the divergence of the 2 proteins, more specifically in the LIR motif containing region.

Supplementary Figure 4 RipA localizes in the cytoplasm of transfected HEK293T cells as punctuated foci. HEK293T cells were transfected using GFP tagged RipA harboring plasmids or vector alone. Twenty four hours post-transfection cells were fixed and the localization of RipA was analyzed using fluorescence microscopy. As shown in the figure, RipA was localized in the cytoplasm and forms punctuate foci (marked by arrows). EGPN-1 vector alone transfected cells were used as a control and show diffuse signal all across the cytoplasm. DAPI was used to mark the nucleus. Scale bar indicates 10 μm.

Supplementary Figure 5 RipA localizes to mitochondria in transfected HEK293T cells. **(A)** Immunofluorescence microscopic images showing mitochondrial colocalization of untagged RipA at 24, 48, and 72 h post-transfection. Vector alone transfected cells (lower panel) showing the distribution of mitochondria and the anti-RipA antibody specificity. Scale bar indicates 10 μm. Anti-RipA antibody was used to probe the localization of RipA. Mitochondrial positions were marked using Mitotracker Deep Red FM dye. DAPI was used to stain the nucleus. A488-conjugated secondary antibody was used for signal detection. Vector alone transfected cells were used as the negative control. **(B)** Analysis of confocal microscopic image for colocalization of RipA and mitochondria using Nikon NIS element software version 5.21.00. Colocalization parameters were shown in the tabulated form at the right of the picture.

Supplementary Figure 6 RipA inhibits apoptosis of macrophage cells. Flow cytometric analysis of early and late apoptotic cells of RipA treated macrophages. HI-treated cells were used as a negative control, whereas staurosporine and ZVAD-FMK served as controls for caspase-dependent apoptosis induction and repression. Briefly, RAW2647.7 cells were seeded in a 6-well-tissue culture plate, after 2 h of adherence at 37°C, the cells were treated with RipA (2 μg/ml), HI RipA (2 μg/ml), 0.1 μM staurosporine, and 2 μM pan caspase inhibitor Z-VAD-FMK. After completion of the treatment, cells were harvested and processed as instruction given by the manufacturer (BD Biosciences, San Jose, USA). Samples were analyzed using BD FACSVerse machine and FlowJo software.

Supplementary Table 1 Details of specific H-bonds at the end of simulations between RipA (Bold) and toll-like receptor (TLR)4.

Supplementary Table 2 Primers used in this study.

Supplementary Table 3 Bacterial strains and plasmids used in this study.

## References

[1] Ernst JD. Macrophage receptors for *Mycobacterium tuberculosis*. Infect Immun. (1998) 66:1277–81. 10.1128/IAI.66.4.1277-1281.1998 9529042 PMC108049

[2] Quesniaux VJ Nicolle DM Torres D Kremer L Guerardel Y Nigou J. Toll-like receptor 2 (TLR2)-dependent-positive and TLR2-independent-negative regulation of proinflammatory cytokines by mycobacterial lipomannans. J Immunol. (2004) 172:4425–34. 10.4049/jimmunol.172.7.4425 15034058

[3] Bowdish DM Sakamoto K Kim MJ Kroos M Mukhopadhyay S Leifer CA. MARCO, TLR2, and CD14 are required for macrophage cytokine responses to mycobacterial trehalose dimycolate and *Mycobacterium tuberculosis*. PLoS Pathog. (2009) 5:e1000474. 10.1371/journal.ppat.1000474 19521507 PMC2688075

[4] Means TK Wang S Lien E Yoshimura A Golenbock DT Fenton MJ. Human toll-like receptors mediate cellular activation by *Mycobacterium tuberculosis*. J Immunol. (1999) 163:3920–7. 10490993

[5] Ku CL Yang K Bustamante J Puel A von Bernuth H Santos OF. Inherited disorders of human Toll-like receptor signaling: immunological implications. Immunol Rev. (2005) 203:10–20. 10.1111/j.0105-2896.2005.00235.x 15661018

[6] Weiss G Schaible UE. Macrophage defense mechanisms against intracellular bacteria. Immunol Rev. (2015) 264:182–203. 10.1111/imr.12266 25703560 PMC4368383

[7] Arora SK Alam A Naqvi N Ahmad J Sheikh JA Rahman SA. Immunodominant *Mycobacterium tuberculosis* protein Rv1507A elicits Th1 response and modulates host macrophage effector functions. Front Immunol. (2020) 11:1199. 10.3389/fimmu.2020.01199 32793184 PMC7385400

[8] Ottenhoff TH Kaufmann SH. Vaccines against tuberculosis: where are we and where do we need to go? PLoS Pathog. (2012) 8:e1002607. 10.1371/journal.ppat.1002607 22589713 PMC3349743

[9] Goletti D Petruccioli E Romagnoli A Piacentini M Fimia GM. Autophagy in *Mycobacterium tuberculosis* infection: a passepartout to flush the intruder out? Cytokine Growth Factor Rev. (2013) 24:335–43. 10.1016/j.cytogfr.2013.01.002 23395260

[10] Nakagawa I Amano A Mizushima N Yamamoto A Yamaguchi H Kamimoto T. Autophagy defends cells against invading group A streptococcus. Science. (2004) 306:1037–40. 10.1126/science.1103966 15528445

[11] Amer AO Swanson MS. Autophagy is an immediate macrophage response to *Legionella pneumophila*. Cell Microbiol. (2005) 7:765–78. 10.1111/j.1462-5822.2005.00509.x 15888080 PMC1584279

[12] Ogawa M Yoshimori T Suzuki T Sagara H Mizushima N Sasakawa C. Escape of intracellular shigella from autophagy. Science. (2005) 307:727–31. 10.1126/science.1106036 15576571

[13] Birmingham CL Smith AC Bakowski MA Yoshimori T Brumell JH. Autophagy controls salmonella infection in response to damage to the salmonella-containing vacuole. J Biol Chem. (2006) 281:11374–83. 10.1074/jbc.M509157200 16495224

[14] Levine B Deretic V. Unveiling the roles of autophagy in innate and adaptive immunity. Nat Rev Immunol. (2007) 7:767–77. 10.1038/nri2161 17767194 PMC7097190

[15] Castillo EF Dekonenko A Arko-Mensah J Mandell MA Dupont N Jiang S. Autophagy protects against active tuberculosis by suppressing bacterial burden and inflammation. Proc Natl Acad Sci USA. (2012) 109:E3168–76. 10.1073/pnas.1210500109 23093667 PMC3503152

[16] Goletti D Petruccioli E Joosten SA Ottenhoff TH. Tuberculosis biomarkers: from diagnosis to protection. Infect Dis Rep. (2016) 8:6568. 10.4081/idr.2016.6568 27403267 PMC4927936

[17] Jo EK. Autophagy as an innate defense against mycobacteria. Pathog Dis. (2013) 67:108–18. 10.1111/2049-632X.12023 23620156

[18] Stanley SA Cox JS. Host-pathogen interactions during *Mycobacterium tuberculosis* infections. Curr Top Microbiol Immunol. (2013) 374:211–41. 10.1007/82_2013_332 23881288

[19] Deretic V Kimura T Timmins G Moseley P Chauhan S Mandell M. Immunologic manifestations of autophagy. J Clin Invest. (2015) 125:75–84. 10.1172/JCI73945 25654553 PMC4350422

[20] Sharma T Grover S Arora NPM Ehtesham NZ Hasnain SE. PGRS domain of Rv0297 of *Mycobacterium tuberculosis* is involved in modulation of macrophage functions to favor bacterial persistence. Front Cell Infect Microbial. (2020) 10:451. 10.3389/fcimb.2020.00451 33042856 PMC7517703

[21] Jagannath C Lindsey DR Dhandayuthapani S Xu Y Hunter RL Jr Eissa NT. Autophagy enhances the efficacy of BCG vaccine by increasing peptide presentation in mouse dendritic cells. Nat Med. (2009) 15:267–76. 10.1038/nm.1928 19252503

[22] Shin DM Jeon BY Lee HM Jin HS Yuk JM Song CH. Mycobacterium tuberculosis eis regulates autophagy, inflammation, and cell death through redox-dependent signaling. PLoS Pathog. (2010) 6:e1001230. 10.1371/journal.ppat.1001230 21187903 PMC3002989

[23] Romagnoli A Etna MP Giacomini E Pardini M Remoli ME Corazzari M. ESX-1 dependent impairment of autophagic flux by Mycobacterium tuberculosis in human dendritic cells. Autophagy. (2012) 8:1357–70. 10.4161/auto.20881 22885411 PMC3442882

[24] Gengenbacher M Nieuwenhuizen N Vogelzang A Liu H Kaiser P Schuerer S. Deletion of nuoG from the vaccine candidate *Mycobacterium bovis* BCG DeltaureC::hly improves protection against tuberculosis. MBio. (2016) 7:e00679–16. 10.1128/mBio.00679-16 27222470 PMC4895111

[25] Yang L Zhang C Zhao Y Zhao N Wu P Zhang H. Effects of *Mycobacterium tuberculosis* mutant strain Hsp16.3 gene on murine RAW 264.7 macrophage autophagy. DNA Cell Biol. (2018) 37:7–14. 10.1089/dna.2016.3599 29068712

[26] Jo EK Yuk JM Shin DM Sasakawa C. Roles of autophagy in elimination of intracellular bacterial pathogens. Front Immunol. (2013) 4:97. 10.3389/fimmu.2013.00097 23653625 PMC3644824

[27] Chai Q Wang X Qiang L Zhang Y Ge P Lu Z. A *Mycobacterium tuberculosis* surface protein recruits ubiquitin to trigger host xenophagy. Nat Commun. (2019) 10:1973. 10.1038/s41467-019-09955-8 31036822 PMC6488588

[28] Shariq M Quadir N Sheikh JA Singh AK Bishai WR Ehtesham NZ. Post translational modifications in tuberculosis: ubiquitination paradox. Autophagy. (2020). 10.1080/15548627.2020.1850009. [Epub ahead of print]. 33190592 PMC8032244

[29] Bhuwan M Arora N Sharma A Khubaib M Pandey S Chaudhuri TK. Interaction of *Mycobacterium tuberculosis* virulence factor RipA with chaperone MoxR1 is required for transport through the TAT secretion system. MBio. (2016) 7:e02259. 10.1128/mBio.02259-15 26933057 PMC4810496

[30] Gupta MN Pandey S Ehtesham NZ Hasnain SE. Medical implications of protein moonlighting. Indian J Med Res. (2019) 149:322–5. 10.4103/ijmr.IJMR_2192_18 31249195 PMC6607823

[31] Truong T Penn BH. An *M. tuberculosis* metabolic enzyme moonlights as an anti-inflammatory effector protein. Cell Host Microbe. (2020) 27:310–2. 10.1016/j.chom.2020.02.012 32164839

[32] Parikh A Kumar D Chawla Y Kurthkoti K Khan S Varshney U. Development of a new generation of vectors for gene expression, gene replacement, and protein-protein interaction studies in mycobacteria. Appl Environ Microbiol. (2013) 79:1718–29. 10.1128/AEM.03695-12 23315736 PMC3591980

[33] Banerjee S Nandyala A Podili R Katoch VM Murthy KJ Hasnain SE. Mycobacterium tuberculosis (Mtb) isocitrate dehydrogenases show strong B cell response and distinguish vaccinated controls from TB patients. Proc Natl Acad Sci USA. (2004) 101:12652–7. 10.1073/pnas.0404347101 15314217 PMC514659

[34] Schneider CA Rasband WS Eliceiri KW. NIH Image to ImageJ: 25 years of image analysis. Nat Methods. (2012) 9:671–5. 10.1038/nmeth.2089 22930834 PMC5554542

[35] Zhang Y. I-TASSER server for protein 3D structure prediction. BMC Bioinformatics. (2008) 9:40. 10.1186/1471-2105-9-40 18215316 PMC2245901

[36] Roy A Kucukural A Zhang Y. I-TASSER: a unified platform for automated protein structure and function prediction. Nat Protoc. (2010) 5:725–38. 10.1038/nprot.2010.5 20360767 PMC2849174

[37] Yang J Yan R Roy A Xu D Poisson J Zhang Y. The I-TASSER Suite: protein structure and function prediction. Nat Methods. (2015) 12:7–8. 10.1038/nmeth.3213 25549265 PMC4428668

[38] Comeau SR Gatchell DW Vajda S Camacho CJ. ClusPro: a fully automated algorithm for protein-protein docking. Nucleic Acids Res. (2004) 32:W96–9. 10.1093/nar/gkh354 15215358 PMC441492

[39] Kozakov D Beglov D Bohnuud T Mottarella SE Xia B Hall DR. How good is automated protein docking? Proteins. (2013) 81:2159–66. 10.1002/prot.24403 23996272 PMC3934018

[40] Kozakov D Hall DR Xia B Porter KA Padhorny D Yueh C. The ClusPro web server for protein-protein docking. Nat Protoc. (2017) 12:255–78. 10.1038/nprot.2016.169 28079879 PMC5540229

[41] Van Der Spoel D Lindahl E Hess B Groenhof G Mark AE Berendsen HJ. GROMACS: fast, flexible, and free. J Comput Chem. (2005) 26:1701–18. 10.1002/jcc.20291 16211538

[42] Hess B Kutzner C van der Spoel D Lindahl E. GROMACS 4: algorithms for highly efficient, load-balanced, and scalable molecular simulation. J Chem Theory Comput. (2008) 4:435–47. 10.1021/ct700301q 26620784

[43] Pronk S Pall S Schulz R Larsson P Bjelkmar P Apostolov R. GROMACS 4.5: a high-throughput and highly parallel open source molecular simulation toolkit. Bioinformatics. (2013) 29:845–54. 10.1093/bioinformatics/btt055 23407358 PMC3605599

[44] Lawrence T. The nuclear factor NF-kappaB pathway in inflammation. Cold Spring Harb Perspect Biol. (2009) 1:a001651. 10.1101/cshperspect.a001651 20457564 PMC2882124

[45] Oeckinghaus A Ghosh S. The NF-kappaB family of transcription factors and its regulation. Cold Spring Harb Perspect Biol. (2009) 1:a000034. 10.1101/cshperspect.a000034 20066092 PMC2773619

[46] Lerner TR Borel S Gutierrez MG. The innate immune response in human tuberculosis. Cell Microbiol. (2015) 17:1277–85. 10.1111/cmi.12480 26135005 PMC4832344

[47] Sippl MJ. Recognition of errors in three-dimensional structures of proteins. Proteins. (1993) 17:355–62. 10.1002/prot.340170404 8108378

[48] Laskowski RA Rullmannn JA MacArthur MW Kaptein R Thornton JM. AQUA and PROCHECK-NMR: programs for checking the quality of protein structures solved by NMR. J Biomol NMR. (1996) 8:477–86. 10.1007/BF00228148 9008363

[49] Wiederstein M Sippl MJ. ProSA-web: interactive web service for the recognition of errors in three-dimensional structures of proteins. Nucleic Acids Res. (2007) 35:W407–10. 10.1093/nar/gkm290 17517781 PMC1933241

[50] Ohto U Fukase K Miyake K Shimizu T. Structural basis of species-specific endotoxin sensing by innate immune receptor TLR4/MD-2. Proc Natl Acad Sci USA. (2012) 109:7421–6. 10.1073/pnas.1201193109 22532668 PMC3358893

[51] Padhi A Pattnaik K Biswas M Jagadeb M Behera A Sonawane A. *Mycobacterium tuberculosis* LprE suppresses TLR2-dependent cathelicidin and autophagy expression to enhance bacterial survival in macrophages. J Immunol. (2019) 203:2665–78. 10.4049/jimmunol.1801301 31619537

[52] Su CC Klenotic PA Bolla JR Purdy GE Robinson CV Yu EW. MmpL3 is a lipid transporter that binds trehalose monomycolate and phosphatidylethanolamine. Proc Natl Acad Sci USA. (2019) 116:11241–6. 10.1073/pnas.1901346116 31113875 PMC6561238

[53] Zandi TA Marshburn RL Stateler PK Brammer Basta LA. Phylogenetic and biochemical analyses of mycobacterial l,d-transpeptidases reveal a distinct enzyme class that is preferentially acylated by meropenem. ACS Infect Dis. (2019) 5:2047–54. 10.1021/acsinfecdis.9b00234 31597040 PMC6910976

[54] Hett EC Chao MC Deng LL Rubin EJ. A mycobacterial enzyme essential for cell division synergizes with resuscitation-promoting factor. PLoS Pathog. (2008) 4:e1000001. 10.1371/journal.ppat.1000001 18463693 PMC2262848

[55] Bah A Lacarriere C Vergne I. Autophagy-related proteins target ubiquitin-free mycobacterial compartment to promote killing in macrophages. Front Cell Infect Microbial. (2016) 6:53. 10.3389/fcimb.2016.00053 27242971 PMC4863073

[56] Lee HJ Venkatarame Gowda Saralamma V Kim SM Ha SE Raha S Lee WS. Pectolinarigenin induced cell cycle arrest, autophagy, and apoptosis in gastric cancer cell via PI3K/AKT/mTOR signaling pathway. Nutrients. (2018) 10:1043. 10.3390/nu10081043 30096805 PMC6115855

[57] Yao R Cooper GM. Requirement for phosphatidylinositol-3 kinase in the prevention of apoptosis by nerve growth factor. Science. (1995) 267:2003–6. 10.1126/science.7701324 7701324

[58] Cowley SC Elkins KL. Immunity to francisella. Front Microbiol. (2011) 2:26. 10.3389/fmicb.2011.00026 21687418 PMC3109299

[59] Tsenova L Bergtold A Freedman VH Young RA Kaplan G. Tumor necrosis factor alpha is a determinant of pathogenesis and disease progression in mycobacterial infection in the central nervous system. Proc Natl Acad Sci USA. (1999) 96:5657–62. 10.1073/pnas.96.10.5657 10318940 PMC21916

[60] Behnsen J Perez-Lopez A Nuccio SP Raffatellu M. Exploiting host immunity: the *Salmonella paradigm*. Trends Immunol. (2015) 36:112–20. 10.1016/j.it.2014.12.003 25582038 PMC4323876

[61] Bekker LG Maartens G Steyn L Kaplan G. Selective increase in plasma tumor necrosis factor-alpha and concomitant clinical deterioration after initiating therapy in patients with severe tuberculosis. J Infect Dis. (1998) 178:580–4. 10.1086/517479 9697749

[62] Marino G Niso-Santano M Baehrecke EH Kroemer G. Self-consumption: the interplay of autophagy and apoptosis. Nat Rev Mol Cell Biol. (2014) 15:81–94. 10.1038/nrm3735 24401948 PMC3970201

[63] Delou JM Biasoli D Borges HL. The Complex link between apoptosis and autophagy: a promising new role for RB. An Acad Bras Cienc. (2016) 88:2257–75. 10.1590/0001-3765201620160127 27991962

[64] Wong PM Feng Y Wang J Shi R Jiang X. Regulation of autophagy by coordinated action of mTORC1 and protein phosphatase 2A. Nat Commun. (2015) 6:8048. 10.1038/ncomms9048 26310906 PMC4552084

[65] Hyttinen JM Niittykoski M Salminen A Kaarniranta K. Maturation of autophagosomes and endosomes: a key role for Rab7. Biochim Biophys Acta. (2013) 1833:503–10. 10.1016/j.bbamcr.2012.11.018 23220125

[66] Xu Y Jagannath C Liu XD Sharafkhaneh A Kolodziejska KE Eissa NT. Toll-like receptor 4 is a sensor for autophagy associated with innate immunity. Immunity. (2007) 27:135–44. 10.1016/j.immuni.2007.05.022 17658277 PMC2680670

[67] Miao J Benomar Y Al Rifai S Poizat G Riffault L Crepin D. Resistin inhibits neuronal autophagy through Toll-like receptor 4. J Endocrinol. (2018) 238:77–89. 10.1530/JOE-18-0096 29773580

[68] Lee JW Nam H Kim LE Jeon Y Min H Ha S. TLR4 (toll-like receptor 4) activation suppresses autophagy through inhibition of FOXO3 and impairs phagocytic capacity of microglia. Autophagy. (2019) 15:753–70. 10.1080/15548627.2018.1556946 30523761 PMC6526818

[69] Behar SM Martin CJ Nunes-Alves C Divangahi M Remold HG. Lipids, apoptosis, and cross-presentation: links in the chain of host defense against *Mycobacterium tuberculosis*. Microbes Infect. (2011) 13:749–56. 10.1016/j.micinf.2011.03.002 21458584 PMC3130819

[70] Deng W Long Q Zeng J Li P Yang W Chen X. Mycobacterium tuberculosis PE_PGRS41 Enhances the Intracellular Survival of M. smegmatis within macrophages via blocking innate immunity and inhibition of host defense. Sci Rep. (2017) 7:46716. 10.1038/srep46716 28440335 PMC5404228

[71] Grover A Izzo AA. BAT3 regulates Mycobacterium tuberculosis protein ESAT-6-mediated apoptosis of macrophages. PLoS ONE. (2012) 7:e40836. 10.1371/journal.pone.0040836 22808273 PMC3396635

[72] Escoll P Buchrieser C. Metabolic reprogramming of host cells upon bacterial infection: why shift to a warburg-like metabolism? FEBS J. (2018) 285:2146–60. 10.1111/febs.14446 29603622

[73] Russell SL Lamprecht DA Mandizvo T Jones TT Naidoo V Addicott KW. Compromised metabolic reprogramming is an early indicator of CD8(+) T cell dysfunction during chronic *Mycobacterium tuberculosis* infection. Cell Rep. 29:3564–79.e5. 10.1016/j.celrep.2019.11.034 31825836 PMC6915325

[74] Cumming BM Addicott KW Adamson JH Steyn AJ. Mycobacterium tuberculosis induces decelerated bioenergetic metabolism in human macrophages. Elife. (2018) 7:e39169. 10.7554/eLife.39169.018 30444490 PMC6286123

[75] Cumming BM Pacl HT Steyn AJC. Relevance of the warburg effect in tuberculosis for host-directed therapy. Front Cell Infect Microbial. (2020) 10:576596. 10.3389/fcimb.2020.576596 33072629 PMC7531540

[76] Mohareer K Medikonda J Vadankula GR Banerjee S. Mycobacterial control of host mitochondria: bioenergetic and metabolic changes shaping cell fate and infection outcome. Front Cell Infect Microbiol. (2020) 10:457. 10.3389/fcimb.2020.00457 33102245 PMC7554303

[77] Ishida R Okamoto T Motojima F Kubota H Takahashi H Tanabe M. Physicochemical properties of the mammalian molecular chaperone HSP60. Int J Mol Sci. 19:489. 10.3390/ijms19020489 29415503 PMC5855711

[78] Blundell TL Gupta MN Hasnain SE. Intrinsic disorder in proteins: relevance to protein assemblies, drug design and host-pathogen interactions. Prog Biophys Mol Biol. (2020) 156:34–42. 10.1016/j.pbiomolbio.2020.06.004 32628954

[79] Gupta MN Alam A Hasnain SE. Protein promiscuity in drug discovery, drug-repurposing and antibiotic resistance. Biochimie. (2020) 175:50–7. 10.1016/j.biochi.2020.05.004 32416199

[80] Ahmed N Dobrindt U Hacker J Hasnain SE. Genomic fluidity and pathogenic bacteria: applications in diagnostics, epidemiology and intervention. Nat Rev Microbiol. (2008) 6:387–94. 10.1038/nrmicro1889 18392032

[81] Kohli S Singh Y Sharma K Mittal A Ehtesham NZ Hasnain SE. Comparative genomic and proteomic analyses of PE/PPE multigene family of *Mycobacterium tuberculosis* H_37_Rv and H_37_Ra reveal novel and interesting differences with implications in virulence. Nucleic Acids Res. (2012) 40:7113–22. 10.1093/nar/gks465 22618876 PMC3424577

[82] Healy C Gouzy A Ehrt S. Peptidoglycan hydrolases RipA and Ami1 are critical for replication and persistence of *Mycobacterium tuberculosis* in the host. MBio. (2020) 11:e03315–19. 10.1128/mBio.03315-19 32127458 PMC7064781

