# Supplementary figures and images for: Mycobacterium tuberculosis RipA Dampens TLR4-Mediated Host Protective Response Using a Multi-Pronged Approach Involving Autophagy, Apoptosis, Metabolic Repurposing, and Immune Modulation
Source: Front Immunol. 2021 Mar 4;12:636644. doi: 10.3389/fimmu.2021.636644 (PMC7969667; doi:10.3389/fimmu.2021.636644)

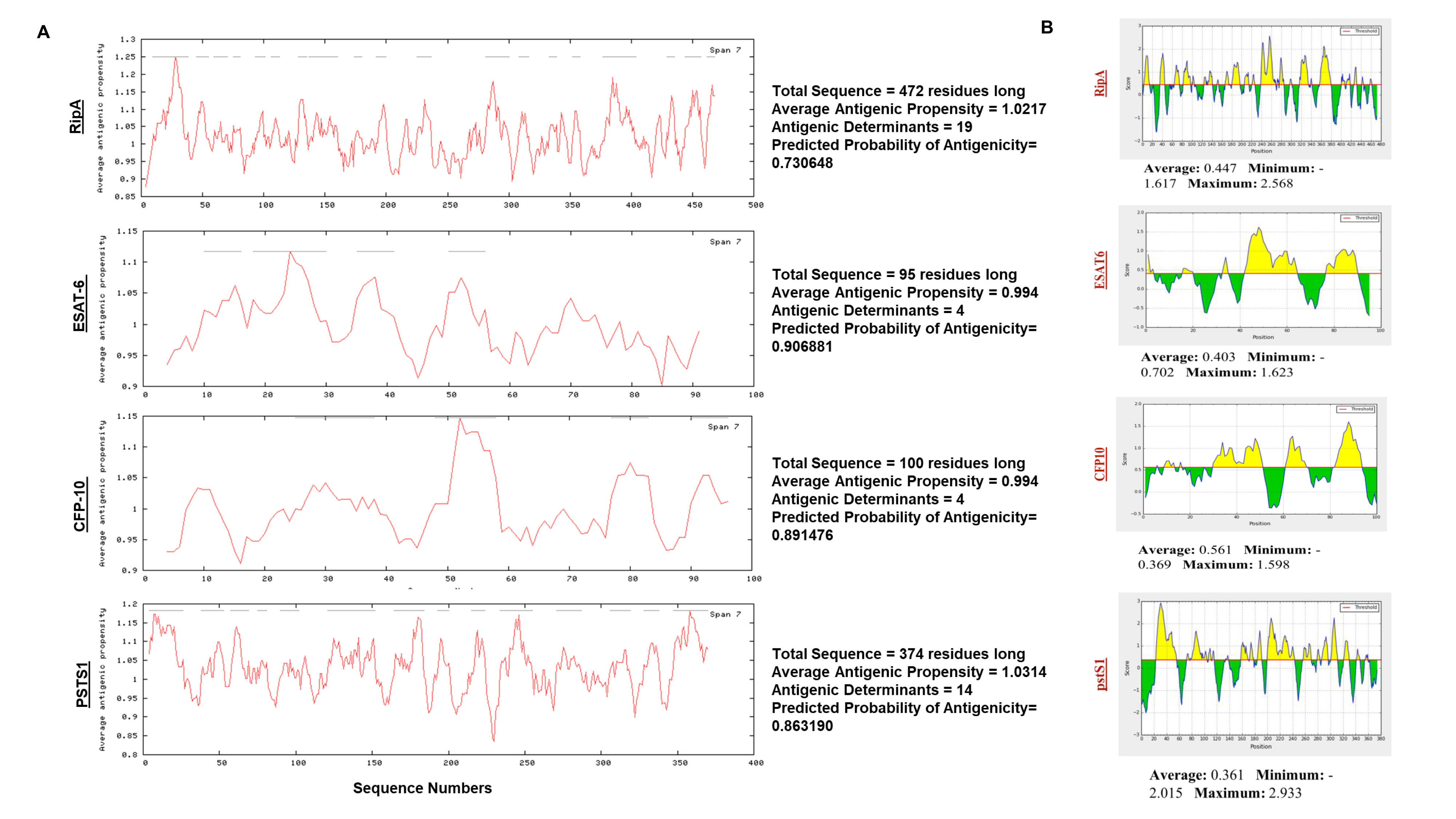

Supplement: Supplementary Figure 1 — Analysis of antigenic potential and B cell epitopes of RipA. (A) The antigenic propensity of RipA was analyzed using the Scratch protein predictor tool and compared with known antigenic molecules of Mycobacterium tuberculosis (M. tb) such as ESAT-6, CFP-10, and PSTS1. As shown in the pictorial representation, RipA displays the comparable antigenic potential to the well-known antigenic molecules of M. tb. (B) B-cell epitope prediction was performed using a B-cell epitope prediction tool. As shown in the figure, RipA harbors sequence stretches that form B-cell epitopes. B-cell epitopes present in RipA were similar to known proteins that have abundant B-cell epitopes such as ESAT-6, CFP-10, and PSTS1. [file Image_1.TIF]

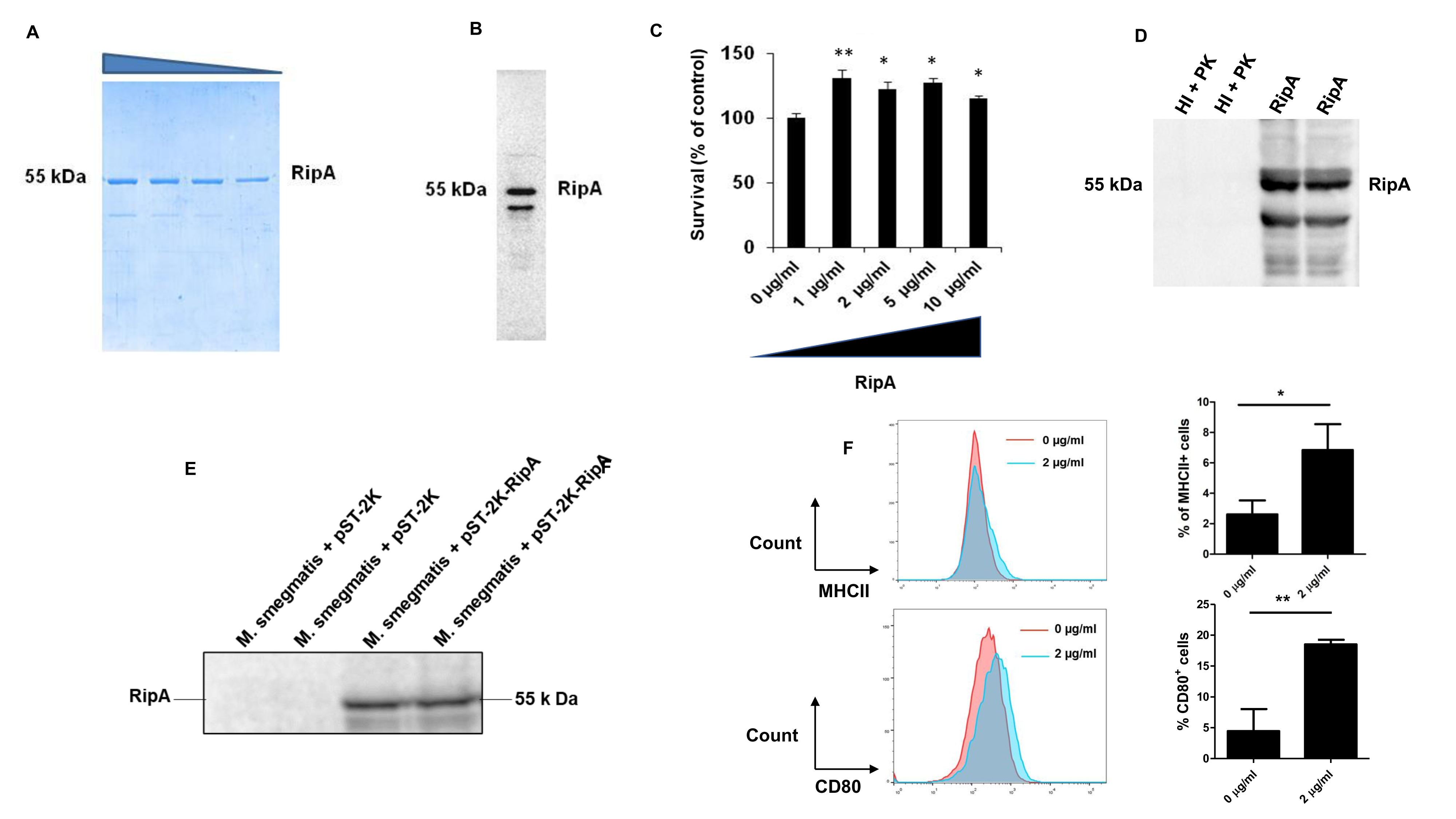

Supplement: Supplementary Figure 2 — Protein purification profile of RipA and its effect on cell survival of RAW264.7 cells. (A) SDS-PAGE profile of affinity-purified RipA. (B) Western blot showing the specificity of the anti-RipA antibody and purity of RipA protein. (C) MTT assay was used to study the effect of RipA on the survival of RAW264.7 cells. RAW264.7 cells were treated with various concentrations of RipA (1, 2, 5, and 10 μg/ml), and cell survival was measured using MTT assay after 24 h of treatment. Untreated cells were used as control. Cell survival in the presence of RipA was analyzed as compared to untreated cells and represented as [%] survival of RAW264.7 cells. (D) Western blot showing proteinase K digested and heat-inactivated (HI) protein samples as well as purified RipA alone probed using anti-RipA antibody. One aliquot of purified RipA was subjected to proteinase K digestion followed by heat inactivation at 100°C for 4 h. Proteinase K digested and HI sample was used as a negative control in each experiment as HI RipA. (E) Western blot showing the expression level of RipA in M. smegmatis transformed with pST-2K vector alone and pST-2K containing ripA. Western blot was performed using anti-RipA polyclonal sera. The enhanced expression of surface markers on THP-1 cells (MHC-II and CD80) at 24 h after treatment with medium alone, and 2 μg/ml RipA. The expression level was determined by FACS analysis using A488 and PE linked monoclonal antibodies, respectively. (F) Graphical representation of MHC-II. Quantitative representation of MHC-II. Graphical representation of CD80. Quantitative representation of CD80 expression on the surface of THP-1 cells. Data are representative of three independent experiments and expressed as means ± SD. *p < 0.05 and **p < 0.01vs. controls. [file Image_2.TIF]

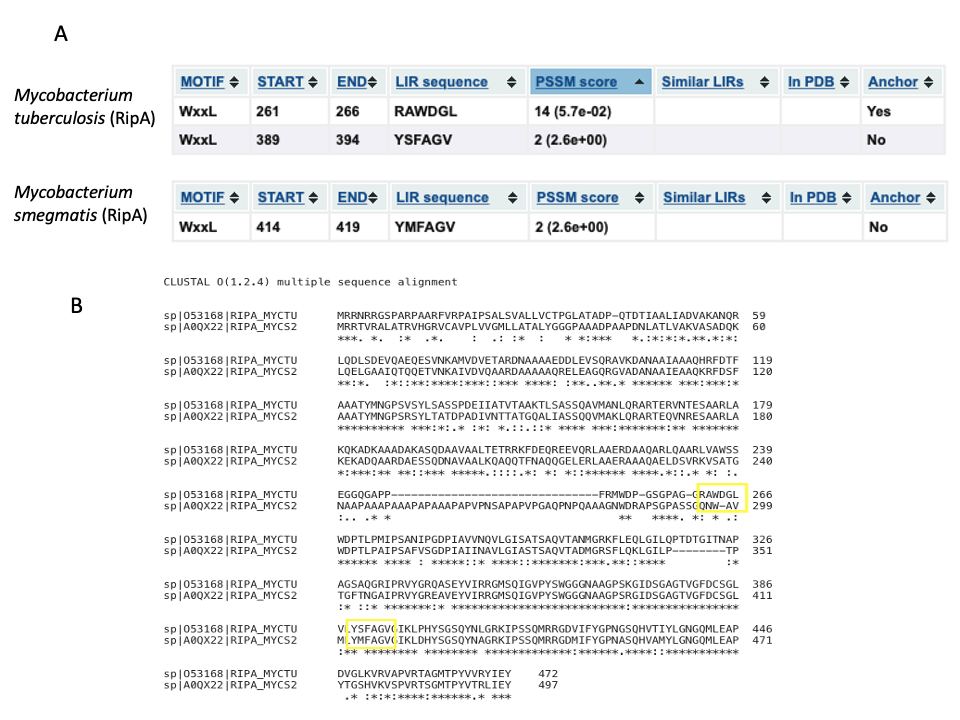

Supplement: Supplementary Figure 3 — M. tb RipA contains two canonical LIR motifs. (A) The RipA protein sequence of M. tb and Mycobacterium smegmatis was analyzed using iLIR web-server (www.repeat.biol.ucy.ac.cy/cgi-bin/iLIR/iLIR_cgi). M. tb RipA was found to encompass a high scoring LC3 interacting motif region (LIR) motif (RAWDGL) that was absent in M. smegmatis RipA. (B) Multiple Sequence Alignment of M. tb RipA and M. smegmatis RipA reveals the divergence of the 2 proteins, more specifically in the LIR motif containing region. [file Image_3.TIF]

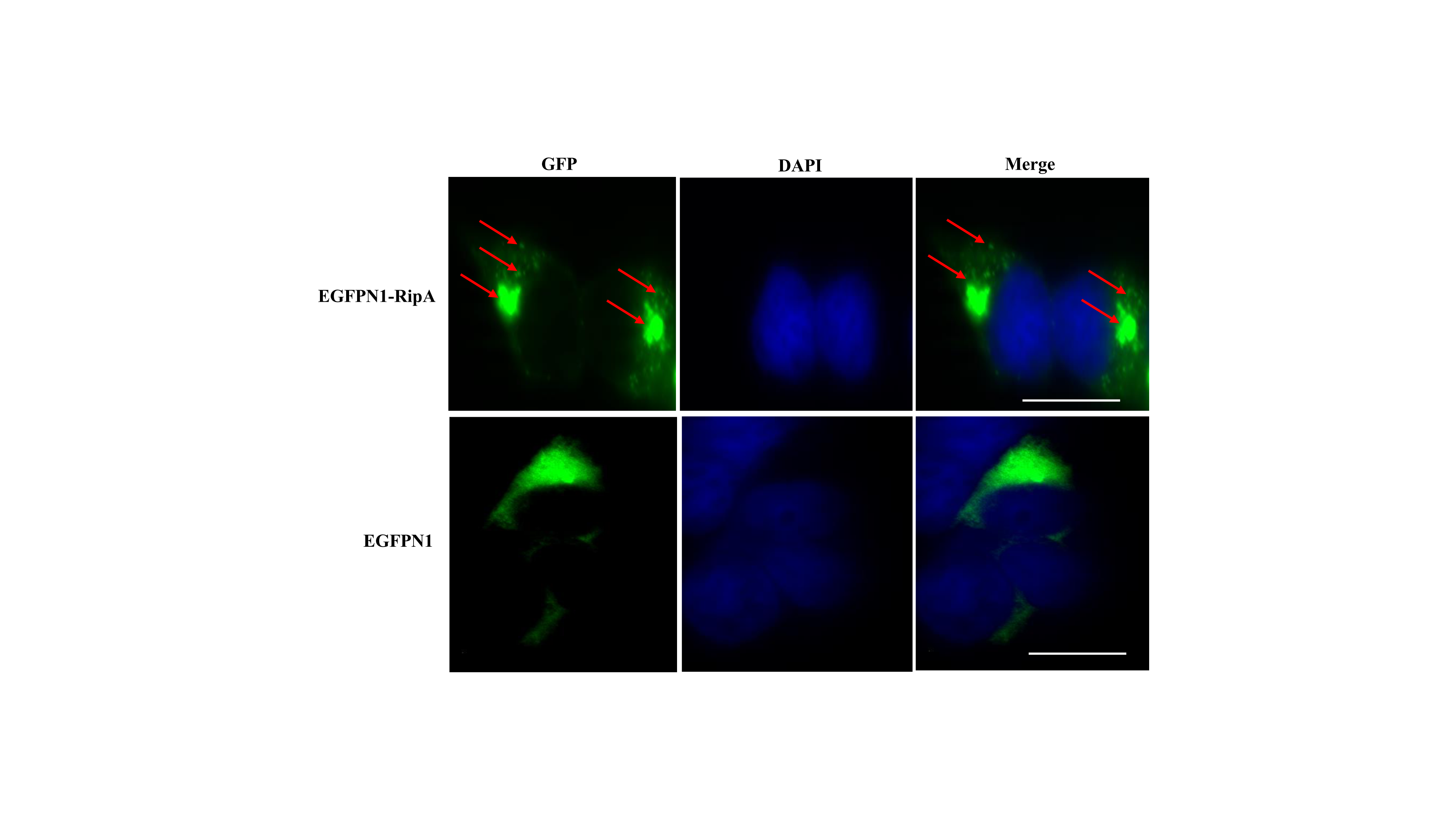

Supplement: Supplementary Figure 4 — RipA localizes in the cytoplasm of transfected HEK293T cells as punctuated foci. HEK293T cells were transfected using GFP tagged RipA harboring plasmids or vector alone. Twenty four hours post-transfection cells were fixed and the localization of RipA was analyzed using fluorescence microscopy. As shown in the figure, RipA was localized in the cytoplasm and forms punctuate foci (marked by arrows). EGPN-1 vector alone transfected cells were used as a control and show diffuse signal all across the cytoplasm. DAPI was used to mark the nucleus. Scale bar indicates 10 μm. [file Image_4.TIF]

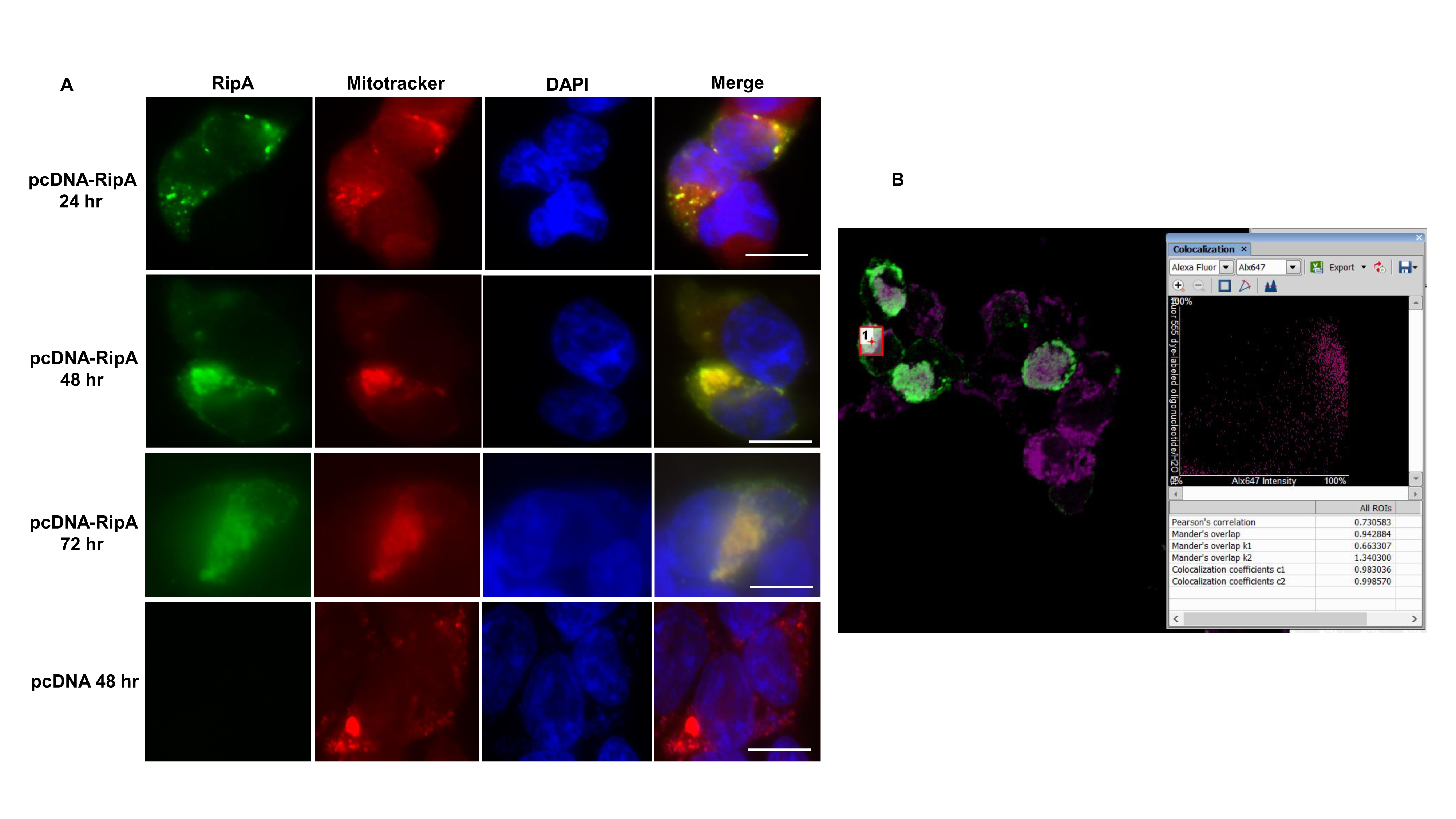

Supplement: Supplementary Figure 5 — RipA localizes to mitochondria in transfected HEK293T cells. (A) Immunofluorescence microscopic images showing mitochondrial colocalization of untagged RipA at 24, 48, and 72 h post-transfection. Vector alone transfected cells (lower panel) showing the distribution of mitochondria and the anti-RipA antibody specificity. Scale bar indicates 10 μm. Anti-RipA antibody was used to probe the localization of RipA. Mitochondrial positions were marked using Mitotracker Deep Red FM dye. DAPI was used to stain the nucleus. A488-conjugated secondary antibody was used for signal detection. Vector alone transfected cells were used as the negative control. (B) Analysis of confocal microscopic image for colocalization of RipA and mitochondria using Nikon NIS element software version 5.21.00. Colocalization parameters were shown in the tabulated form at the right of the picture. [file Image_5.TIF]

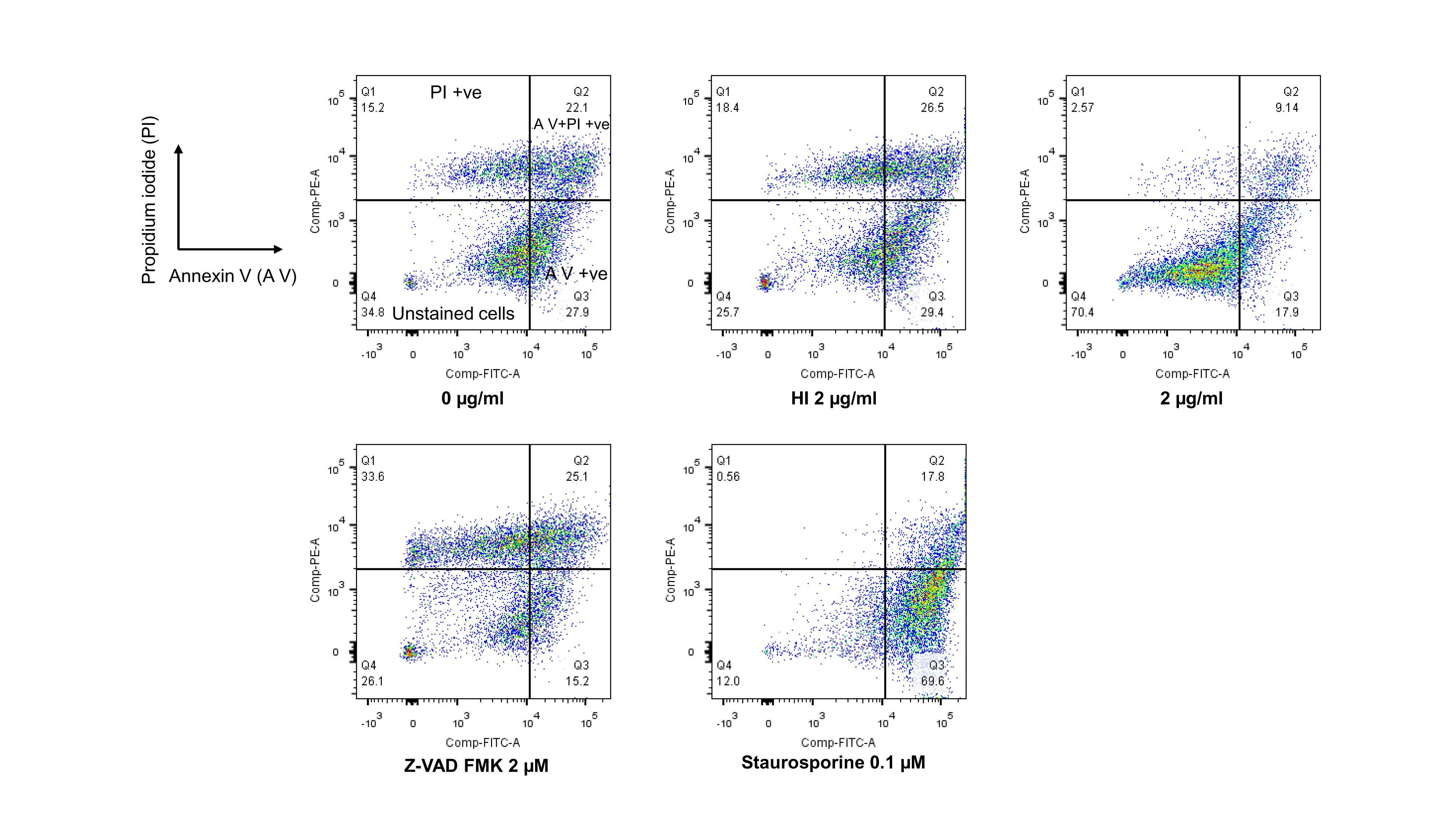

Supplement: Supplementary Figure 6 — RipA inhibits apoptosis of macrophage cells. Flow cytometric analysis of early and late apoptotic cells of RipA treated macrophages. HI-treated cells were used as a negative control, whereas staurosporine and ZVAD-FMK served as controls for caspase-dependent apoptosis induction and repression. Briefly, RAW2647.7 cells were seeded in a 6-well-tissue culture plate, after 2 h of adherence at 37°C, the cells were treated with RipA (2 μg/ml), HI RipA (2 μg/ml), 0.1 μM staurosporine, and 2 μM pan caspase inhibitor Z-VAD-FMK. After completion of the treatment, cells were harvested and processed as instruction given by the manufacturer (BD Biosciences, San Jose, USA). Samples were analyzed using BD FACSVerse machine and FlowJo software. [file Image_6.TIF]
