# Supplementary material for: Mycobacterium tuberculosis RipA Dampens TLR4-Mediated Host Protective Response Using a Multi-Pronged Approach Involving Autophagy, Apoptosis, Metabolic Repurposing, and Immune Modulation
Source: Front Immunol. 2021 Mar 4;12:636644. doi: 10.3389/fimmu.2021.636644 (PMC7969667; doi:10.3389/fimmu.2021.636644)
Supplement: Supplementary Table 1 — Details of specific H-bonds at the end of simulations between RipA (Bold) and toll-like receptor (TLR)4. [file Table_1.DOCX]

**GLY 281** – ASN 155

**GLN 289** – ASP 83

**ASP 373** – GLU 229

**ALA 376** – GLU 286

**GLY 355** – HIS 401

**SER 352** – THR 473

**ARG 348** – ASP 548

**ARG 419** – THR 546

**ARG 340** – ASN 572

**GLN 341** – THR 574 / ASN 575

**TYR 338** – ASN 575

**TYR 472** – GLN 603

**ASN 428** – GLN 80
