# Supplementary material for: Mycobacterium tuberculosis RipA Dampens TLR4-Mediated Host Protective Response Using a Multi-Pronged Approach Involving Autophagy, Apoptosis, Metabolic Repurposing, and Immune Modulation
Source: Front Immunol. 2021 Mar 4;12:636644. doi: 10.3389/fimmu.2021.636644 (PMC7969667; doi:10.3389/fimmu.2021.636644)
Supplement: Supplementary Table 2 — Primers used in this study. [file Table_2.DOCX]

| **Primer name** | **Nucleotide sequence (5’ 3’)** | **Comment** |
| --- | --- | --- |
| pET28a-RipAF | ATCATATGAGACGGAATCGCCGTGGCTCG | Cloning of *ripA* into pET28a |
| pET28a-RipAR | ATGCGGCCGCGTACTCGATGTATCGGACCAC |  |
| EGFPN1-RipAF | ATGAATTCATGAGACGGAATCGCCGTGGC | Cloning of *ripA* into EGFPN1 |
| EGFPN1-RipAR | TTGGTACCGTACTCGATGTATCGGAC |  |
| pcDNA3.1-RipAF | GAAAGCTTGCCGCCATGGGACGGAATCGCCGT | Cloning of *ripA* into pcDNA3.1+ |
| pcDNA3.1-RipAR | ATGCGGCCGCGTACTCGATGTATCGGACCCAC |  |
