# Supplementary material for: Mycobacterium tuberculosis RipA Dampens TLR4-Mediated Host Protective Response Using a Multi-Pronged Approach Involving Autophagy, Apoptosis, Metabolic Repurposing, and Immune Modulation
Source: Front Immunol. 2021 Mar 4;12:636644. doi: 10.3389/fimmu.2021.636644 (PMC7969667; doi:10.3389/fimmu.2021.636644)
Supplement: Supplementary Table 3 — Bacterial strains and plasmids used in this study. [file Table_3.DOCX]

| Strains and plasmids | Description | Source/Ref |
| --- | --- | --- |
| **Strains** |  |  |
| *E. coli* |  |  |
| BL21(DE3) | *F^–^ ompT gal dcm lon hsdS_B_*(r_B_^-^ m_B_^-^) *λ*(DE3) [*lacI lacUV5-*T7 *gene 1 ind1 sam7 nin5*]) | Invitrogen |
| DH5α | *F^-^ [ϕ80dΔlacZM15] Δ(lacZYA-argF)U169 deoR recA1 endA1 hsd R17 glnV44 thi-1 gyrA96 relA1* | Gibco-BRL |
| Mycobacterium |  |  |
| *M. smegmatis* mc^2^ 155 | *ept-1* | (1) |
| **Plasmids** |  |  |
| pET28a | Km^r^, His tag protein expression vector | Novagen |
| EGFPN1 | Km^r^, GFP tag protein expression vector for mammalian cells | Novagen |
| pcDNA3.1+ | Ap^r^, Protein expression vector for mammalian cells | Invitrogen |
| pET28a-RipA | Km^r^, full length coding region of *ripA* cloned in pET28a | This study |
| EGFPN1-RipA | Km^r^, full length coding region of *ripA* cloned in EGFPN1 | This study |
| pcDNA3.1-RipA | Ap^r^, full length coding region of *ripA* cloned in pcDNA3.1+ | This study |

1. **Snapper SB, Melton RE, Mustafa S, Kieser T, Jacobs WR, Jr.** 1990. Isolation and characterization of efficient plasmid transformation mutants of *Mycobacterium smegmatis*. Mol Microbiol **4:**1911-1919.
